# *Maclura tinctoria* as a Modulator of Oxidative Stress and Inflammatory Responses

**DOI:** 10.3390/ijms27125504

**Published:** 2026-06-18

**Authors:** Eduarda Pires Costa, Allan Rodrigues Pires, Mariáurea Matias Sarandy, Romulo Dias Novaes, Geraldo Célio Brandão, Joao Paulo Viana Leite, Iorrana Vieira Salustiano, Debora Esposito, Reggiani Vilela Gonçalves

**Affiliations:** 1 Department of General Biology, Federal University of Viçosa, Viçosa 36570-900, MG, Brazil; eduarda.costa@ufv.br (E.P.C.); allan.pires@ufv.br (A.R.P.); 2 Department of Animal Biology, Federal University of Viçosa, Viçosa 36570-900, MG, Brazil; mariaureasarandy@gmail.com; 3 Department of Structural Biology, Federal University of Alfenas, Alfenas 37130-001, MG, Brazil; romulo.novaes@unifal-mg.edu.br; 4 School of Pharmacy, Federal University of Ouro Preto, Ouro Preto 35400-000, MG, Brazil; celiobrandao@ufop.edu.br; 5 Department of Biochemistry and Molecular Biology, Federal University of Viçosa, Viçosa 36570-900, MG, Brazil; jpvleite@ufv.br (J.P.V.L.); iovieirasalus@gmail.com (I.V.S.); 6 Plants for Human Health Institute, Animal Science, North Carolina Research Campus, North Carolina State University, Kannapolis, NC 28081, USA; daesposi@ncsu.edu

**Keywords:** *Maclura tinctoria*, antioxidant, oxidative stress, inflammation, cytokines

## Abstract

Oxidative stress plays a central role in the progression of inflammatory and degenerative diseases, highlighting the need for natural compounds with antioxidant and anti-inflammatory potential. This study investigated the biological activity of the dichloromethane extract of *Maclura tinctoria* leaves (DcMt), which was selected for further analyses after initial screening demonstrated superior antioxidant activity compared with the hexane extract (HxMt). Antioxidant capacity was evaluated by DPPH and FRAP assays, while cellular effects were assessed in RAW 264.7 macrophages through analyses of viability, cytokine gene expression, COX-2 modulation, catalase activity, and cell migration. LC-DAD-ESI-MS profiling revealed a well-defined chromatographic composition dominated by four major constituents, which were isolated and structurally elucidated by NMR and MS as prenylated flavonoids, several of which are reported here for the first time in *M. tinctoria*. DcMt exhibited strong antioxidant activity and preserved cell viability under oxidative stress, with optimal effects at 25 µg/mL, accompanied by increased catalase activity. The extract modulated inflammatory markers by increasing IL-10 and IL-6 gene expression, maintaining IL-1β levels, and regulating COX-2 expression. In addition, DcMt promoted macrophage migration, further supporting its potential role in modulating inflammatory responses. Importantly, all biological assays were performed using the crude extract, and the contribution of individual compounds remains to be further investigated. These findings support *M. tinctoria* as a promising source of bioactive compounds with antioxidant and immunomodulatory potential.

## 1. Introduction

The increasing demand for natural alternatives for treating various inflammatory diseases has fueled research into plant extracts for their antioxidant, anti-inflammatory, and regenerative properties [1,2,3]. Oxidative stress, which results from an imbalance between reactive oxygen species (ROS) production and the cellular antioxidant defense, plays a crucial role in the onset and progression of chronic diseases [4,5]. The release of free radicals and ROS can lead to lipid, protein, and DNA oxidation, compromising cellular integrity and triggering inflammatory and degenerative processes [6]. During inflammation, ROS and RNS are produced by macrophages in the process known as the respiratory burst [7]. Excess reactive species act as signaling molecules, promoting the up-regulation of pro-inflammatory cytokines and the down-regulation of anti-inflammatory cytokines, resulting in chronic inflammation and altered cellular functions. In this context, oxidative stress also impairs cell migration and proliferation and affects the expression and function of inflammatory mediators. Therefore, a positive feedback loop between oxidative stress and inflammation can be observed, known as the OxInflammation process [8]. This interconnected process highlights the importance of simultaneously evaluating redox balance and inflammatory signaling when investigating potential therapeutic agents. Cells rely on both enzymatic and non-enzymatic antioxidant defense systems to counteract oxidative damage. Enzymatic antioxidants include superoxide dismutase (SOD), catalase (CAT), and glutathione peroxidase (GPx), whereas non-enzymatic defenses comprise molecules such as glutathione (GSH), vitamin C, and vitamin E [9]. However, under conditions of excessive oxidative stress, these protective mechanisms may become overwhelmed, underscoring the need for compounds that can modulate both oxidative stress and inflammation [10].

Inflammation plays a central role in the progression of many diseases, with cytokines such as IL-2, IL-6, IL-10, and IL-1β playing pivotal roles in immune regulation [11]. IL-2 is primarily involved in lymphocyte activation and proliferation, linking innate and adaptive immune responses. Although not classically associated with macrophages, IL-2 can indirectly influence macrophage activity by modulating T-cell responses and cytokine signaling, thereby contributing to the regulation of the inflammatory environment, while IL-6 and IL-1β are associated with pro-inflammatory signaling and amplification of immune responses [12]. In contrast, IL-10 acts as an anti-inflammatory mediator that helps regulate excessive immune activation [13]. In addition, COX-2, an inducible enzyme upregulated during inflammation, contributes to prostaglandin production, further intensifying inflammatory signaling and, consequently, the OxInflammation process [14]. Together, these mediators are closely associated with the regulation and resolution of inflammation, making them relevant targets for evaluating the immunomodulatory effects of natural compounds.

Natural products rich in bioactive compounds, such as polyphenols and flavonoids, have been extensively studied for their antioxidant and anti-inflammatory properties. The therapeutic potential of plant extracts is often linked to their ability to modulate the antioxidant and anti-inflammatory responses of immune system cells [15]. In this context, macrophages are widely used in vitro studies to assess the effects of bioactive compounds on cell viability, proliferation, and migration [16], as they play a central role in the OxInflammation process by producing reactive species and cytokines in response to stress stimuli. Among these models, RAW 264.7 macrophages are particularly suitable due to their well-characterized response to oxidative stress and inflammatory signaling, making them a reliable system for evaluating immunomodulatory effects. This cellular model provides valuable insights into the mechanisms through which natural compounds influence physiological and pathological processes [17]. Beyond their antioxidant properties, certain plant extracts have demonstrated protective effects against oxidative stress-induced cellular damage. This protective capability is attributed to their ability to enhance endogenous defense mechanisms and mitigate the harmful effects of ROS on cellular structures [18]. Investigating these effects can expand our understanding of the broader therapeutic potential of plant-based compounds in preventing or managing inflammatory/oxidative stress-related conditions.

Our pharmaceutical bioprospecting group has evaluated the biological activity of hundreds of plant extracts from the Brazilian Atlantic Forest against various biological targets, identifying promising cytotoxic [19] and antibacterial [20] compounds. One of our target plant species is *Maclura tinctoria* (L.) D. Don ex Steud. (Moraceae), popularly known as “amora-do-mato” or “taiúva”. In traditional Brazilian medicine, preparations from the bark and stem exudate of *M. tinctoria* are used as cicatrizants and analgesics to treat back pain and also as anti-inflammatories after dental extraction [21]. Other reported uses include antirheumatic infusions and treatments for urinary tract infections, sore throat, gout, and syphilis [22]. These uses, particularly those related to wound healing and inflammation, suggest potential effects on processes such as cell migration and cytokine modulation, indicating a broad spectrum of pharmacological potential. This aligns with scientific findings on the species’ bactericidal activity, particularly a prenylated flavonoid-enriched fraction that has demonstrated effectiveness against *Staphylococcus aureus* [23].

Previous studies reported the chemical identifications of prenylated flavonoids, chalcones, and xanthones isolated from *M. tinctoria* [24]. Phytochemical studies have revealed that this species is rich in phenolic compounds, which exhibit potent antioxidant, antimicrobial, anti-inflammatory, and cytoprotective activities [25]. These bioactive flavonoids have been reported to modulate key signaling pathways involved in oxidative stress, immune response, and cell homeostasis. Therefore, this study aimed to: (i) investigate the effects of *M. tinctoria* extract on the interplay between oxidative stress and inflammation and their influence on cell migration and proliferation; (ii) define an appropriate extraction solvent and evaluate the antioxidant and anti-inflammatory capacity of the plant leaves; and (iii) explore the role of key inflammatory mediators, including IL-1β, IL-6, and IL-10, in the regulation of the OxInflammation process following extract exposure.

## 2. Results

### 2.1. Collection of Plant Material and Extraction

The extract with the highest yield was dichloromethane (DcMt), at 3.47% (*w*/*w*), followed by hexane (HxMt), at 2.53% (*w*/*w*). These solvents were selected based on their intermediate (dichloromethane) and low (hexane) polarity, allowing the extraction of different classes of metabolites, such as lipophilic compounds and moderately polar constituents. HxMt exhibited an oily appearance at room temperature, with a yellowish coloration. DcMt appeared in an amorphous form with a greenish coloration. Based on both extraction yield and subsequent biological activity (data shown below), DcMt was selected for further phytochemical and biological analyses.

### 2.2. DPPH Radical Scavenging

**Figure 1:**
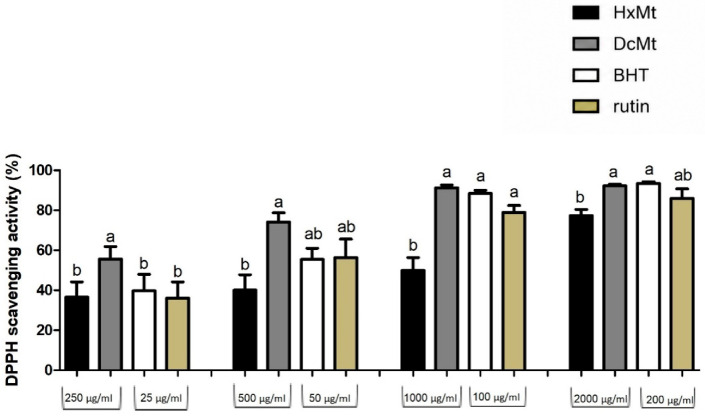
Assessment of the antioxidant capacity of *M. tinctoria* leaf extracts through the DPPH radical scavenging technique (2,2-diphenyl-1-picrylhydrazyl). Different letters indicate statistically significant differences between groups (*p* < 0.05, ANOVA followed by Tukey’s post hoc test). Data are presented as mean ± standard error of the mean (SEM).

The DcMt extract exhibited a higher DPPH scavenging activity than HxMt at all tested concentrations (Figure 1), confirming its superior antioxidant potential. Although quantitative parameters such as IC_50_ were not determined, a clear concentration-dependent increase in activity was observed for both extracts. The results were compared with the commercial antioxidants rutin and BHT. While DcMt required concentrations approximately 10-fold higher to achieve similar levels of radical scavenging, it reached comparable or, at higher concentrations, slightly higher activity than BHT and surpassed rutin. These findings indicate that despite its lower potency, DcMt exhibits significant antioxidant capacity.

### 2.3. Cell Viability

**Figure 2:**
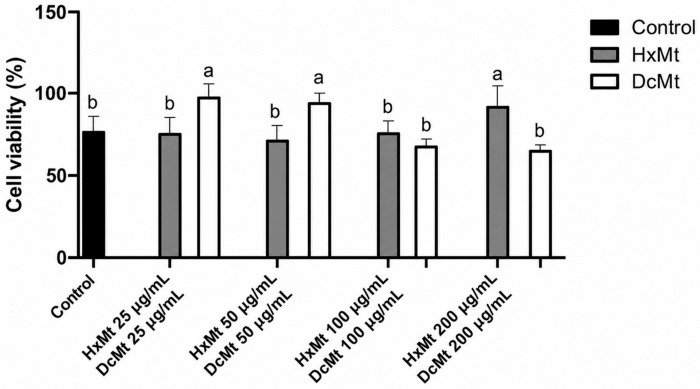
Effect of *M. tinctoria* leaf extracts on macrophage cell viability. Control = culture medium and water; DcMt = dichloromethane extract; HxMt = hexane extract. Different letters indicate statistically significant differences between groups (*p* < 0.05, ANOVA followed by Tukey’s post hoc test). Data are presented as mean ± standard error of the mean (SEM).

The impact of HxMt and DcMt extracts on cell viability was assessed to evaluate their cytotoxic potential in this cell line. DcMt at 25 μg/mL and 50 μg/mL resulted in higher cell viability than the other groups (Figure 2), indicating a possible cytoprotective effect at these concentrations. In contrast, HxMt showed higher cell viability than DcMt at 200 μg/mL. Although both extracts maintained cell viability across the tested concentrations, DcMt demonstrated more pronounced effects at lower doses, suggesting greater biological activity under these conditions. It is important to note that the observed increase in viability may reflect cytoprotection rather than direct stimulation of cell proliferation. Based on its overall performance, particularly at lower concentrations, DcMt was selected for subsequent biological and phytochemical analyses.

### 2.4. Ferric Reducing Antioxidant Power (FRAP) Assay

**Figure 3:**
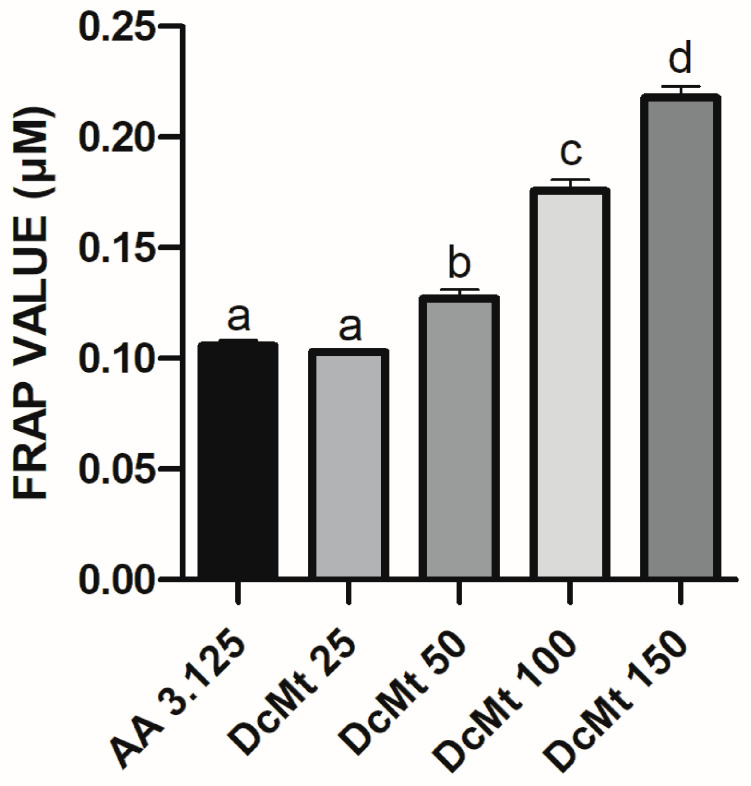
Reducing Antioxidant Power (FRAP) assay. The FRAP assay measured antioxidant capacity and expressed the result as absorbance at 593 nm. AA 3.125 refers to the ascorbic acid control (3.125 µg/mL), while DcMt 25, DcMt 50, DcMt 100, and DcMt 150 correspond to increasing sample concentrations (25, 50, 100, and 150 µg/mL, respectively). Different letters indicate statistically significant differences between groups (*p* < 0.05, ANOVA followed by Tukey’s post hoc test). Data are presented as mean ± standard error of the mean (SEM).

A dose-dependent increase in FRAP values was observed, indicating a higher antioxidant potential at higher concentrations, with the highest FRAP value recorded at 150 µg/mL. This result reflects the extract’s electron-donating ability (Figure 3).

### 2.5. Assessment of RAW264.7 Macrophage Cell Viability After Induction of Oxidative Stress with H_2_O_2_

**Figure 4:**
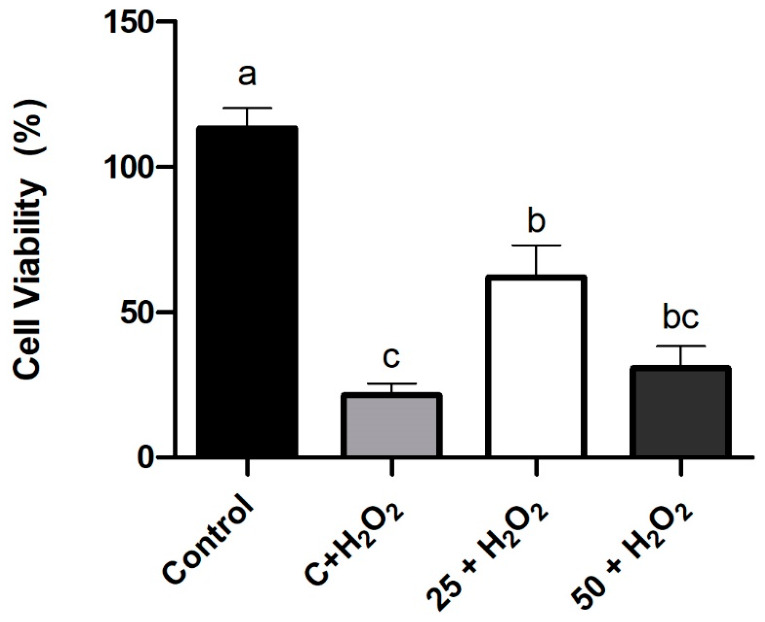
Evaluation of macrophage cell viability after induction of oxidative stress with H_2_O_2_. Positive Control = culture medium + 0.4% DMSO; Negative control C+ H_2_O_2_ = culture medium + 0.4% DMSO + 2 mM H_2_O_2_; 50 + H_2_O_2_ and 25 + H_2_O_2_ are relative to the concentration of DcMt extract in μg/mL + 2 mM H_2_O_2_. Data are expressed as mean ± SEM. Different letters indicate statistically significant differences between groups (*p* < 0.05, ANOVA followed by Tukey’s post hoc test).

H_2_O_2_ exposure significantly reduced cell viability (C+ H_2_O_2_ group). Treatment with the extract improved cell viability in a concentration-dependent manner, with a significant increase at 25 μg/mL relative to the H_2_O_2_-treated control group, and 50 μg/mL also enhanced cell viability. This result suggests that lower concentrations may be more effective at preserving cellular metabolic activity under oxidative stress, possibly reflecting a more favorable balance between antioxidant activity and cytotoxicity (Figure 4).

### 2.6. Evaluation of Catalase with RAW264.7 Macrophages

**Figure 5:**
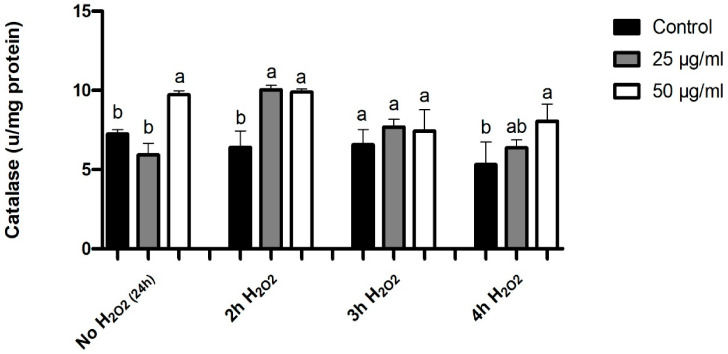
The activity of catalase in macrophage homogenates obtained from cells treated with DcMt extract for 24 h at concentrations of 25 and 50 µg/mL, followed by exposure or not to H_2_O_2_ for different durations (2, 3, and 4 h). Data are expressed as mean ± SEM. Different letters indicate statistically significant differences between groups (*p* < 0.05, ANOVA followed by Tukey’s post hoc test).

Catalase activity was measured in macrophage homogenates. Cells treated with DcMt extract at 50 μg/mL under non-stressed conditions (without H_2_O_2_ for 24 h) exhibited higher catalase activity than the control and 25 μg/mL groups (*p* < 0.05). After 2 h of H_2_O_2_ exposure, both 25 and 50 μg/mL treatments increased catalase activity compared to the H_2_O_2_-treated control (*p* < 0.05), with the 25 μg/mL group showing the highest enzymatic activity at this time point. No significant differences were observed at 3 h (Figure 5). After 4 h of exposure, the 50 μg/mL treatment maintained higher catalase activity than the control (*p* < 0.05), but was lower than that observed at 2 h. These findings suggest a time- and concentration-dependent modulation of catalase activity, possibly reflecting an adaptive antioxidant response of macrophages to oxidative stress.

### 2.7. Scratch Assay

**Figure 6:**
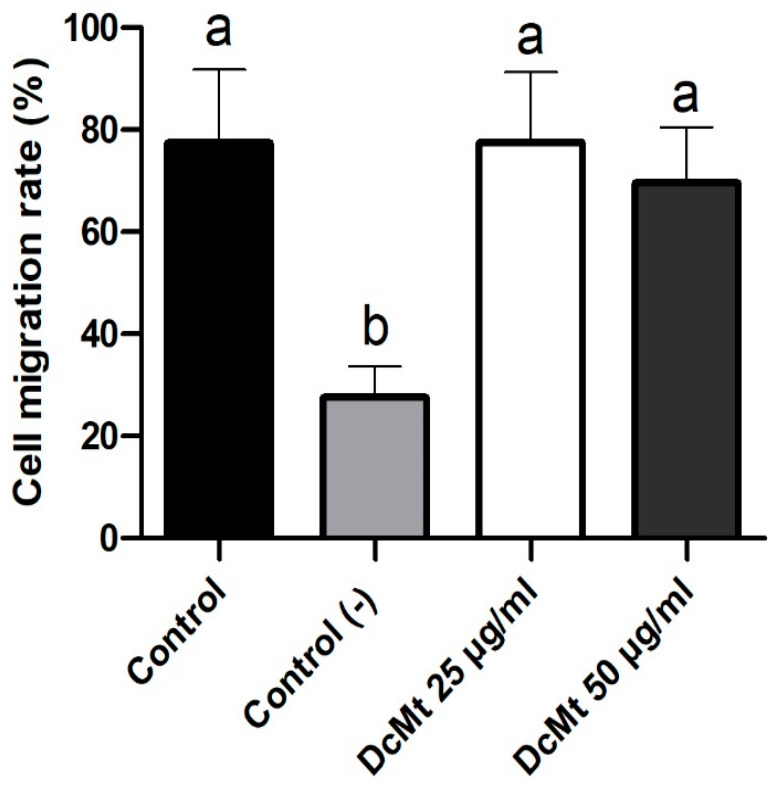
Cell migration rate after treatment with DcMt extract. Control = culture medium; Control (−) = culture medium + 0.4% DMSO. Data are expressed as mean ± SEM. Different letters indicate statistically significant differences between groups (*p* < 0.05, ANOVA followed by Tukey’s post hoc test).

H_2_O_2_ exposure significantly reduced macrophage migration, decreasing the migration rate to approximately 30% compared to the control (80%). Treatment with DcMt extract at 25 and 50 μg/mL restored cell migration to approximately 75% and 70%, respectively, indicating a recovery of macrophage migratory capacity under oxidative stress conditions (Figure 6). This assay reflects macrophage migration behavior rather than wound-healing processes.

### 2.8. mRNA Extraction and Gene Expression Analysis by Real-Time PCR

**Figure 7:**
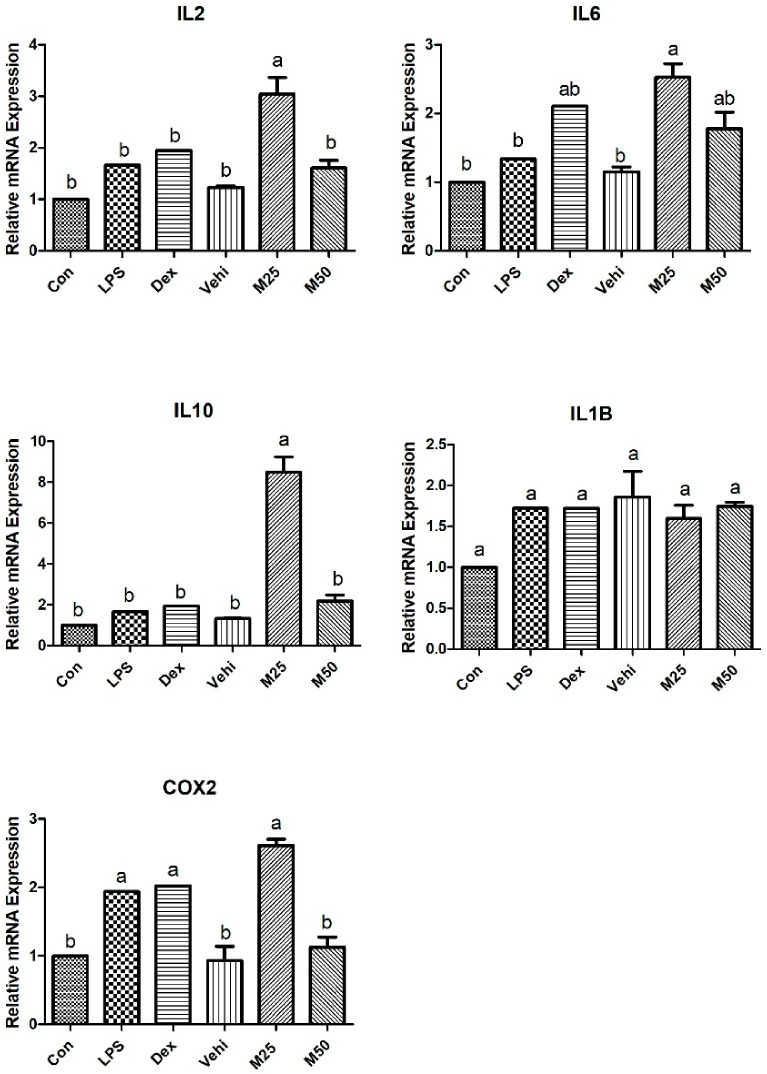
Relative mRNA expression of inflammatory and anti-inflammatory markers in different experimental conditions. The mRNA expression levels of IL-2, IL-6, IL-10, IL-1β, and COX-2 were evaluated by qPCR in other treatment groups: control (Con), LPS-stimulated (LPS), dexamethasone-treated (Dex), vehicle-treated (Vehi), DcMt extract at 25 μg/mL (M25), and DcMt extract at 50 μg/mL (M50). Data are expressed as mean ± SEM. Different letters indicate statistically significant differences between groups (*p* < 0.05, ANOVA followed by Tukey’s post hoc test).

The gene expression analysis revealed significant differences among the experimental groups. IL-2 and IL-10 expression levels were significantly upregulated in the DcMt extract at 25 μg/mL group compared to all other conditions (*p* < 0.05). Expression was also significantly higher in the DcMt extract at 25 μg/mL than in the other groups. There were no statistical differences in IL-1β expression among the groups. COX2 expression was significantly elevated in the DcMt extract at the 25 μg/mL group, while DcMt extract at 50 μg/mL and Dex groups showed lower expression levels (Figure 7).

### 2.9. Isolation and Identification of Compounds from DcMt Extract

**Figure 8:**
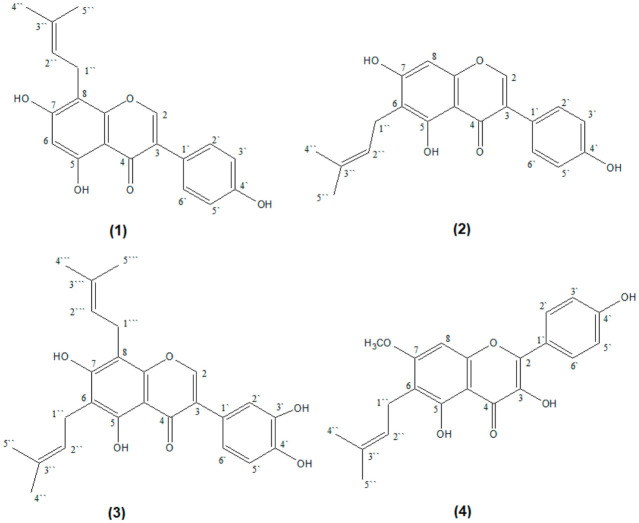
Prenylated flavonoids isolated from the dichloromethane extract of *Maclura tinctoria* leaves (DcMt).

LC-DAD-ESI-MS analysis of DcMt revealed a well-defined chromatographic profile dominated by four major peaks at retention times of 6.07, 6.35, 6.93, and 7.03 min (Figure S1). These peaks corresponded to the predominant constituents of the extract and guided the subsequent purification procedures. Phytochemical screening indicated the presence of flavonoids and triterpenes/steroids in both HxMt and DcMt extracts, while DcMt also tested positive for phenolic compounds. The purification process involved silica gel column chromatography followed by semipreparative HPLC, leading to the isolation of four major prenylated flavonoids: compound **1** (83.8 mg, lupiwighteone), compound **2** (64.7 mg, wighteone), compound **3** (163.2 mg, 6,8-diprenylorobol), and compound **4** (16.6 mg, 3,4′,5-trihydroxy-6-(3″-methylbut-2″-enyl)-7-methoxyflavonol) (Figure 8) (Table 1). The direct correspondence between the major chromatographic peaks and the isolated compounds confirms that these metabolites define the chemical profile of DcMt. These prenylated flavonoids are widely reported in the literature for their antioxidant and anti-inflammatory properties, which may be associated with the biological effects observed in this study. Detailed physicochemical and spectroscopic data are provided in the Supplementary Materials.

## 3. Discussion

The growing interest in plant-derived therapeutics has stimulated the investigation of medicinal species with potential anti-inflammatory properties. In this context, *M. tinctoria*, traditionally used in Brazilian folk medicine, represents a promising source of bioactive compounds. Therefore, the present study investigated its antioxidant and anti-inflammatory activities, and the findings are discussed considering current evidence regarding the modulation of inflammatory pathways by plant-derived phytochemicals. In this context, our study was structured to investigate its antioxidant activity, cytoprotective effects, macrophage behavior, and cytokine modulation, which are key processes involved in oxidative stress and inflammation. Specifically, we explored its antioxidant capacity to counteract oxidative stress, as well as its effects on cell proliferation and migration, which are essential for tissue repair and regeneration.

LC-DAD-ESI-MS analysis of DcMt revealed a chromatographic profile with four major peaks corresponding to the predominant constituents of the extract. Previous phytochemical investigations by our research group demonstrated that the dichloromethane fraction of *M. tinctoria* is enriched in prenylated flavonoids, with five compounds structurally characterized by UPLC-DAD and LC-DAD-ESI-MS analyses [19]. The predominance of prenylated flavonoids observed in the present study is consistent with these findings and confirms the phytochemical reproducibility of the dichloromethane extract. Furthermore, the enhanced biological activity previously reported for this fraction highlights the relevance of these compounds as potential contributors to the biological effects observed in the present study.

Four compounds were isolated and identified from DcMt. The compounds **1** and **2**, with retention times of 6.07 and 6.35 min, showed ^1^H NMR and ^13^C NMR spectra suggesting that they are two isomers, with signals corresponding to aromatic hydrogens characteristic of an AA′BB′ system. Doublets at *J* 8.5 Hz, δ 7.3 and 6.9 ppm are assigned to hydrogens of ring B (Figure S1). A singlet at δ 7.94 (H-2) confirmed the structural isoflavone skeleton. The presence of the 3,3-dimethylallyl group [δ 1.6 and 1.8 (3 H, singlet), 3.4 (2 H, multiplet), and 5.2 (1 H, multiplet) was detected in compound **1** (Figure S2, Supplementary Materials). Furthermore, ^13^C-NMR spectra and DEPT indicate the presence of 13 signals associated with the isoflavone chemical structure, a prenyl group with two olefinic carbons (122.0 and 132.0), two methyl groups (25.6 and 17.6), and one methylene carbon (21.4) (Figures S3 and S4). The location of the 3,3-dimethylallyl group was assigned to C-8 of ring A by the HMBCs of H-2″ (δ 5.2) with C-8 (δ 105.4) (Figure S5). Based on the data above, compound **1** was identified as 5,7,4′-trihydroxy-8-[3′-methylbut-2′-en-1-yl]-isoflavone (lupiwighteone). This compound was previously isolated from the species *Lupinus luteus* and *Glycyrrhiza glabra* [26]. This is the first report of compound **1** in *M. tinctoria*. Besides that, ^1^H-NMR, ^13^C-NMR data, and HMBC confirmed that compounds **1** and **2** are isomers with a 3,3-dimethylallyl group attributed with C-6 in the last one. The correlations between H-2″ (5.27) and C-6 (112.0), and between H-1″ (1.79) and C-5 (159.2), support the prenyl group position. Thus, compound **2** was identified as 5,7,4′-trihidroxi-6-[3″-metilbut-2″-enil]-isoflavona (wighteone) (Figures S7–S11). This substance has already been isolated from *M. tinctoria* fruits [27].

Compound **3**, with a retention time of 6.93 min, is an isoflavone with two prenyl units. The prenyl group was assigned to C-6 to the correlation between H-1″ (3.32) and C-5 (157.3), and between H-2″ (5.09) and C-6 (110.5). The second prenyl group was assigned to C-8, confirmed by the corresponding H-1 (δ 3.32) and C-9 (δ 153.6) signals. Thus, compound **3** was identified as 5,7,3′,4′-tetrahydroxy-6-[3″-methylbut-2″-enyl]-8-[3″′-methylbut-2″-enyl]-isoflavone. This compound is known as 6,8-diprenylorobol (Figures S12–S17). Its presence has been reported in another species of the Moraceae family, *Cudrania tricuspidata* [28]. However, this is the first record for the species *M. tinctoria*.

The ^1^H-NMR and ^13^C-NMR spectra of compound **4** show signals for the methoxy group, observed at 3.74 (1H), and 55.4 (^13^C). The group 3,3-dimethylallyl [δ 1.6 and δ 1.7 (3 H, singlet), δ 3.36 (2 H, multiplet) and δ 5.15 (1 H, multiplet)] was also detected (Figures S18 and S19). The linkage of the methoxy group in C-7 was correlated with 3.7 of methoxy protons with δ 161.6 of C-7. The prenyl group was associated with H-1 (δ 3.35), C-6 (δ 109.5), and C-5 (δ 157.8). Therefore, compound **4**, which is also unpublished in *M. tinctoria*, was identified as 3,5,4′-trihydroxy-6-[3″-methylbut-2″-enyl]-7-methoxy-flavonol. The presence of the prenylated flavonoids lupiwighteone, wighteone, 6,8-diprenylorobol, and 3,5,4′-trihydroxy-6-[3″-methylbut-2″-enyl]-7-methoxy-flavonol in *Maclura tinctoria*, with three of them being reported for the first time in the species, reinforces its pharmacological potential (Figures S20–S23). The literature reports selective cytotoxic activity of lupiwighteone and anti-inflammatory properties of 6,8-diprenylorobol [28], supporting the relevance of these metabolites and encouraging further investigation into their biological applications.

The antioxidant capacity of DcMt was initially evaluated using the DPPH radical-scavenging assay, in which the extract showed notable activity at concentrations of 250 and 500 µg/mL, suggesting it contains bioactive molecules that efficiently neutralize free radicals, comparable to or even slightly exceeding the performance of standard antioxidants such as BHT and rutin. Interestingly, previous investigations with wood and bark extracts of *M. tinctoria* have shown that even at lower concentrations (around 25 µg/mL), these extracts can rapidly quench over 50% of the DPPH radicals within the first 5 min, hinting at a remarkable antioxidant potential possibly linked to phenolic compounds, including prenylated flavones and isoflavones [24]. Together, these findings highlight the promise of DcMt as a nuanced yet potent source of natural antioxidant agents, meriting further exploration in the context of managing oxidative stress and inflammation.

Our findings revealed that the DcMt extract preserved cell viability more effectively at concentrations of 25 and 50 µg/mL. In contrast, the HxMt extract required a higher concentration (200 µg/mL) to achieve a comparable effect, suggesting lower biological potency. Notably, at concentrations of 100 and 200 µg/mL, the DcMt extract exhibited cytotoxicity, potentially due to a pro-oxidant action associated with elevated antioxidant levels. This observation aligns with the concept that, under certain conditions, phenolic compounds may reduce iron and copper ions, enhancing the formation of peroxide-free radicals and posing an oxidative risk [29]. Similarly, studies on *Morus nigra* L., another member of the Moraceae family, have demonstrated that while lower concentrations of its extracts can promote cell proliferation, higher doses may lead to cytotoxic effects. For instance, an aqueous methanolic extract of *M. nigra* leaves exhibited dose-dependent inhibition of HeLa cancer cells, with an IC_50_ of 56.0 µg/mL, indicating significant cytotoxicity at higher concentrations [30]. These findings underscore the importance of optimizing dosage when evaluating the therapeutic potential of bioactive compounds from plant extracts. Therefore, the DPPH radical-scavenging assay and the macrophage viability test yielded superior results for DcMt, reinforcing its higher antioxidant potential and biological efficacy. Consequently, DcMt was selected for further in vitro experiments to explore its better therapeutic potential. Since initial tests indicated that the DcMt extract exhibited higher antioxidant activity in both the DPPH assay and preserving cell viability, we decided to perform the FRAP assay exclusively with this extract. Additionally, in the FRAP assay, we used lower concentrations than those tested in the DPPH assay, bringing them closer to the concentrations used in the cellular assays. This approach enabled a stronger correlation between the extract’s chemical antioxidant capacity and the biological effects observed in cells, reinforcing the selection of DcMt as the most promising extract for therapeutic investigations. The results obtained in the FRAP assay demonstrated a dose-dependent increase in the antioxidant capacity of the DcMt extract, suggesting that the presence of phenolic compounds and prenylated flavonoids in the extract may contribute to its ferric ion-reducing activity, as evidenced by its ability to inhibit DPPH radicals. This behavior can be explained by the fact that in a chemical system like FRAP, a higher extract concentration results in more antioxidant molecules available for electron donation, thereby enhancing its antioxidant activity [31]. This trend was also observed in the DPPH assay, where the DcMt extract exhibited a high capacity to neutralize free radicals, comparable to that of reference antioxidants such as BHT and rutin. Both assays (FRAP and DPPH) assess the extract’s antioxidant potential in chemical systems, independent of cellular mechanisms, thereby explaining the observed dose–response correlation. Although both assays are based on chemical systems, FRAP primarily reflects electron-transfer capacity, whereas DPPH evaluates radical-scavenging activity, indicating complementary antioxidant mechanisms.

The next step was to understand the cells’ behavior after exposure to the stressful agent associated with the extract. After stressing the cells with H_2_O_2_ exposure, we observed that 25 µg/mL provided the highest cell viability under oxidative stress. This apparent discrepancy may be associated with hormesis, in which moderate doses of bioactive compounds induce beneficial adaptive responses, whereas higher doses may exert pro-oxidant or cytotoxic effects [32]. At higher concentrations, DcMt may intensify the reduction of transition metals, thereby increasing the formation of reactive oxygen species (ROS) via Fenton reactions and consequently causing detrimental effects on cells. Thus, while in the FRAP and DPPH assays, a higher antioxidant content results in greater reducing power and a higher capacity to neutralize free radicals, in cellular models, the modulation of the antioxidant response occurs more complexly, being influenced by the cells’ ability to balance oxidative stress without exceeding a critical threshold of cellular damage [33]. These findings reinforce the importance of determining optimal therapeutic doses of *M. tinctoria* extract to ensure antioxidant efficacy without compromising cell viability. Although the antioxidant activity of *M. tinctoria* has been demonstrated in chemical assays, studies investigating its effects on macrophage viability under oxidative stress remain lacking.

Analyzing the cytoprotective effect of the extract against H_2_O_2_-induced stress, we observed that at a concentration of 25 µg/mL, it neutralizes and protects cells from damage caused by free radicals and reactive oxygen species generated by H_2_O_2_ exposure. Phytochemical compounds may reduce stress by triggering a beneficial adaptive response that prepares cells for a prompt reaction to future stress [34]. This process, called hormesis, may explain the observed effect [35]. Although H_2_O_2_ is one of the most important products in the initial stage of tissue repair, its levels need to be controlled to prevent damage to cellular processes, which are mediated by enzymatic systems and antioxidant molecules [36]. To further investigate the antioxidant potential of DcMt extracts, we examined their ability to regulate intracellular antioxidant defenses. Using H_2_O_2_ as a stressor, we quantified catalase (CAT) activity, as uncontrolled ROS production and deficiencies in this enzyme’s defense mechanisms can impair cell proliferation and migration [37]. Our catalase analysis revealed that both extract concentrations exhibited notable antioxidant activity, with a particularly strong effect observed at 50 µg/mL. This suggests that the extract promotes the rapid decomposition of hydrogen peroxide into water and oxygen, effectively mitigating oxidative stress. Similar findings have been reported in studies with human fibroblasts, in which exposure to proanthocyanidins from persimmon peel extract for 24 h, followed by H_2_O_2_ treatment for 2 h, increased catalase activity compared with controls [38]. These results highlight the crucial role of bioactive compounds, such as prenylated flavonoids, anthocyanins, and proanthocyanidins, in modulating ROS production. By promoting cellular adaptation, enhancing cytoprotection, and preventing apoptosis, these compounds help maintain cellular homeostasis [39].

Macrophage migration is widely used as an in vitro model to investigate cellular responses under inflammatory conditions. These cells exhibit phagocytic capacity and act as antigen-presenting cells, as well as sources of growth factors and biochemical mediators that regulate immune responses [40]. Disruption of the cell monolayer results in the loss of cell–cell contact, triggering a coordinated response. The subsequent release of growth factors and cytokines stimulates macrophage migration, mimicking conditions observed in inflammatory environments. Previous in vitro studies with other cell types, such as fibroblasts, have shown that *Dicranopteris linearis* extract can induce the secretion of pro-inflammatory cytokines, enhancing cell motility and responsiveness to chemotactic signals [41]. Our results demonstrated that the DcMt extract significantly accelerated macrophage migration, suggesting that its effects may be linked to the activation of chemotaxis-related pathways. However, this effect should be interpreted with caution, as increased migration does not necessarily indicate a beneficial or detrimental outcome; rather, it reflects modulation of cellular responsiveness to inflammatory stimuli. Based on these findings, we propose that DcMt treatment modulates cell recruitment, a fundamental process in the dynamics of inflammation. Furthermore, regarding inflammatory markers, the results of this study highlight the significant therapeutic potential of the DcMt extract at 25 μg/mL, as it modulates the inflammatory response in a balanced manner. The marked increase in IL-10 expression observed in the group treated with the DcMt extract at 25 μg/mL suggests that this extract may contribute to inflammation resolution and tissue repair, as IL-10 is a key anti-inflammatory cytokine that suppresses excessive immune activation and promotes tissue regeneration, thereby fostering a more regulated and potentially regenerative environment [42]. This profile may help explain the effects observed in cytoprotection and proliferation assays and contribute to the modulation of cellular responses, such as migration. By reducing inflammatory stress and favoring cellular repair mechanisms, the DcMt extract at 25 μg/mL could become a valuable therapeutic agent for inflammatory and degenerative conditions.

In addition to IL-10, the DcMt extract at 25 μg/mL significantly increased IL-6 expression, a cytokine with a dual role in inflammation. Although IL-6 is traditionally associated with the acute phase of inflammation, it also contributes to tissue regeneration and cellular defense mechanisms. IL-6 can directly induce IL-10 production, thereby contributing to the resolution of inflammation. As demonstrated by Andres-Hernando [43], in vitro, the addition of IL-6 to splenocytes increased IL-10 production in CD4+ T cells, B cells, and macrophages, and in vivo data further support its role in enhancing IL-10 production during anti-inflammatory responses. Within the macrophage polarization paradigm, this cytokine profile may indicate a shift toward an M2-like phenotype. M2 macrophages are associated with anti-inflammatory functions, tissue repair, and resolution of inflammation, and are characterized by increased IL-10 production. In this context, Braune et al. [44] demonstrated that IL-6 can act as a Th2-associated cytokine, promoting macrophage polarization toward the M2 phenotype and stimulating their local proliferation in adipose tissue. This increased activity of M2-type macrophages is essential for modulating the immune response and preventing excessive tissue damage. However, it is important to consider that IL-6 is also a well-established pro-inflammatory mediator. The ability of the DcMt extract at 25 μg/mL to increase IL-6 expression without a corresponding increase in IL-1β, a potent pro-inflammatory cytokine typically associated with the M1 phenotype, may suggest a controlled inflammatory response without progression to a chronic inflammatory state. This balanced modulation between pro- and anti-inflammatory signals may be particularly advantageous in therapeutic contexts, as excessive IL-1β levels are often implicated in chronic inflammatory diseases [45]. Nevertheless, this interpretation should be approached with caution, and further investigation is required to better understand the role of IL-6 in this context and to directly confirm macrophage polarization markers.

Another important finding supporting the therapeutic potential of the DcMt extract is its impact on COX-2 expression. The increase in COX-2 levels observed in the 25 μg/mL DcMt extract group may indicate an initial inflammatory response, but this enzyme is also involved in prostaglandin-mediated pathways that regulate cell migration and proliferation [46]. The controlled elevation of COX-2 suggests that the DcMt extract at 25 μg/mL does not trigger an excessive inflammatory response but rather supports essential cellular processes for wound healing and tissue regeneration. This effect may be particularly relevant in conditions where controlled inflammation is necessary to initiate the healing process, such as tissue injuries and degenerative diseases [47]. The observed increase in cell migration assays may be directly linked to COX-2-mediated pathways, further reinforcing the role of the DcMt extract at 25 μg/mL in facilitating repair mechanisms. However, as COX-2 is also a classical pro-inflammatory marker, these findings should be interpreted with caution and require further investigation.

Finally, the significant upregulation of IL-2 following treatment with DcMt at 25 μg/mL suggests an immunomodulatory effect that may contribute to the regulation of macrophage function and inflammatory signaling. IL-2 is crucial for T cell proliferation and immune system modulation, which may contribute to a more dynamic immune response. The ability of the DcMt extract at 25 μg/mL to increase IL-2 expression suggests it may help maintain immune homeostasis and promote an environment conducive to repair and regeneration [48]. Our findings reveal that the DcMt extract at a concentration of 25 μg/mL exhibits a remarkable ability to finely modulate inflammatory responses, exerting both protective and regenerative effects. Interestingly, its dual action appears to balance the activation of essential inflammatory pathways with the resolution of inflammation, a process potentially mediated by the upregulation of IL-10. This unique capability positions DcMt as a promising candidate for therapeutic applications in inflammatory diseases, cell migration, and degenerative disorders.

The phytochemical composition of *M. tinctoria* provides important clues regarding the mechanisms underlying the observed effects. Previous studies have identified the presence of prenylated flavonoids, which are known to modulate key signaling pathways, including Nrf2 and NF-κB, involved in redox balance and inflammatory responses. Although these pathways were not directly assessed in the present study, the biological effects observed are consistent with the activities previously reported for prenylated flavonoids. It is important to note that the individual contribution of each compound was not evaluated, primarily due to the limited availability of isolated standards and the technical challenges in obtaining sufficient quantities of pure compounds from plant material. Furthermore, it should be considered that plant extracts often exert their biological effects through synergistic interactions among multiple constituents, and therefore the activity observed for the DcMt extract may not be attributable to a single compound. Future studies involving bioassay-guided fractionation and the evaluation of isolated compounds, as well as their potential synergistic interactions, are necessary to further elucidate the specific contributions of prenylated flavonoids.

## 4. Materials and Methods

### 4.1. Collection of Plant Material and Extraction

The leaves of *M. tinctoria* were collected in the Atlantic Forest fragment at coordinates 20°48′07″ S and 42°51′31″ W, belonging to the Federal University of Viçosa (UFV), Minas Gerais, Brazil. The plant name was checked using The Plant List on 23 March 2012. The sample exsiccate was deposited in the UFV herbarium (voucher number VIC 40,269). The leaves were dried in a ventilated oven at a temperature below 40 degrees and then pulverized in a knife mill. The dried leaves (550 g) were first exhaustively extracted with hexane by percolation. The plant residue was then subjected to a second exhaustive extraction using dichloromethane under the same conditions. Both extracts were dried using a vacuum rotary evaporator and subsequently lyophilized to ensure complete removal of all solvents, including dichloromethane. All biological assays were performed using the dried extracts, ensuring the absence of residual solvent. In this way, the hexane (HxMt, 13.9 g) and dichloromethane (DcMt, 19.1 g) extracts were obtained. After production of the dry extracts, they were stored in sealed glass containers at −20 °C, protected from light.

### 4.2. DPPH Radical Scavenging

To assess the antioxidant capacity of the *M. tinctoria* leaf extract, the DPPH radical scavenging technique was used [25]. The methodology measures the inhibitory capacity of compounds against the action of free radicals by the decrease in absorbance in the reaction medium [49]. Antioxidant activity is evaluated by the reduction of the DPPH radical, forming diphenyl-picryl-hydrazine, a yellow-colored compound, in a reaction that stabilizes after 30 min from its initiation. The assay relied on preparing a standard curve of DPPH absorbance from HxMt and DcMt extracts (2, 1, 0.500, 0.250 mg/mL) diluted in methanol. BHT (butylated hydroxytoluene) and rutin were used as standards/controls for antioxidant activity at concentrations of 200, 100, 50, and 25 µg/mL, also diluted in methanol. Different concentration ranges were used because BHT and rutin are purified antioxidant compounds with higher radical-scavenging potency, whereas plant extracts are complex mixtures that require higher concentrations to elicit a measurable antioxidant response. For each extract concentration or BHT/rutin, 180 µL of DPPH (120 µM) was added, and the reaction mixtures were incubated at room temperature (approximately 25 °C) in the dark for 30 min to allow complete radical stabilization. Subsequently, the absorbance was measured at 517 nm using a microplate reader. The experiments were performed in triplicate. The percentage of DPPH radical inhibition was calculated using the formula below (1):

(1) $$DPPH\;INHIBITION\;(\%)=\frac{Abs\;DPPH/methanol\;80\%-Abs\;extract\times 100}{Abs\;DPPH/methanol\;80\%}$$

### 4.3. Analysis of Cell Viability Using RAW264.7 Macrophages

Cell proliferation tests performed on RAW 264.7 macrophages were evaluated by the 3-(4,5-dimethylthiazol-2-yl)-2,5-diphenyltetrazolium bromide (MTT) reduction method [50]. The macrophages were plated at a density of 2 × 10^4^ cells per well in 200 μL of DMEM supplemented with 10% fetal bovine serum (FBS) in 96-well plates and incubated in a 37 °C, 5% CO_2_ humidified incubator for 24 h. After this incubation period, the supernatant was removed, and the HxMt and DcMt extracts were added at concentrations of 200, 100, 50, and 25 μg/mL. The extracts were dissolved in DMSO and diluted in culture medium to achieve a final DMSO concentration of 0.4% in all treated wells. Two controls were performed: the first containing culture medium and dimethyl sulfoxide (DMSO) at a concentration of 0.4%, and the second containing only culture medium. The cells were incubated in the incubator (37 °C and 5% CO_2_) for 22 h. After this period, 50 μL of supernatant from each well was withdrawn, and 50 μL of MTT solution (0.5 mg/mL) was added. The cells were incubated again for an additional 2 h. Subsequently, formation of formazan crystals was observed, and 90 μL of supernatant from each well was removed, followed by adding 100 μL of DMSO to each well to stop the reaction. The absorbance was measured on an ELISA Multiskan FC Microplate Reader (Thermo Labsystems, Franklin, MA, USA) set to 570 nm. The experiments were performed in triplicate.

### 4.4. Ferric Reducing Antioxidant Power (FRAP) Assay

The total antioxidant capacity of the extracts was determined using the Ferric Reducing Antioxidant Power (FRAP) assay, as previously described [31], with TPTZ (2,4,6-Tris(2-pyridyl)-s-triazine) as the substrate. This method is based on the reduction of a ferric 2,4,6-tripyridyl-s-triazine complex (Fe^3+^ + -TPTZ) to its ferrous form (Fe^2+^) + -TPTZ. Samples (10 μL) of the DcMt extract at concentrations of 25 µg/mL, 50 µg/mL, 100 µg/mL, and 150 µg/mL were added to the FRAP solution (190 μL), which consisted of 25 mL of acetate buffer (300 mmol/L, pH 3.6), 2.5 mL of TPTZ reagent (10 mmol/L), and 2.5 mL of FeCl_3_·6H_2_O solution (20 mmol/L). The increase in absorbance at 593 nm was measured to determine the reduction of the Fe^3−^ TPTZ complex by antioxidants in the samples. The reducing capacity was quantified using a standard curve prepared from serial dilutions of FeSO_4_·7H_2_O starting at one mmol/L. The results were expressed as FRAP values. A concentration of 3125 µg/mL of Ascorbic acid was used as the reference standard for comparison. The experiments were performed at least in triplicate, and the results were expressed as FRAP values (µg/mL).

### 4.5. Hydrogen Peroxide (H_2_O_2_)-Mediated Oxidative Stress Analyses

To induce oxidative stress, cells were exposed to H_2_O_2_. Macrophages were plated at a density of 2 × 10^4^ cells/well in a 24-well plate containing DMEM culture medium supplemented with 10% fetal bovine serum (FBS). The cultures were incubated in a CO_2_ incubator (37 °C and 5% CO_2_) for 24 h. A concentration curve of H_2_O_2_ was constructed (0.125; 0.25; 0.5; 1.0; and 2.0 mM) to determine the dose that would result in a reduction in cell viability by up to 92% after 24 h of exposure. A control with only culture medium was included. Cell viability assessment was performed using the MTT method described in Section 4.3.

### 4.6. Evaluation of the Cell Viability of RAW264.7 Macrophages After Induction of Oxidative Stress with H_2_O_2_

After determining the H_2_O_2_ concentration with a deleterious effect of up to 92%, we conducted the cell viability test using DcMt to assess the protective effect of *M. tinctoria* extract. From this point onwards, all analyses were performed using only the DcMt extract, as it yielded better results than HxMt in previous studies. Macrophages were plated at a density of 2 × 10^4^ cells/well in a 96-well plate containing DMEM culture medium supplemented with 10% fetal bovine serum (FBS). The cultures were incubated overnight in a CO_2_ incubator (37 °C and 5% CO_2_). For treatment, macrophages were cultured in DMEM supplemented with 10% FBS and 25 or 50 μg/mL of the extract. They were incubated in the incubator (37 °C and 5% CO_2_) for 24 h and subsequently exposed to the DcMt extract at concentrations that showed better performance in previous analyses (50 and 25 μg/mL). After 24 h, the cells were exposed to 2 mM H_2_O_2_ for 2 h. Cell viability was assessed using the MTT assay described in Section 4.3.

### 4.7. Evaluation of Catalase with RAW264.7 Macrophages

Macrophages were plated (1 × 10^6^ cells/well) in 6-well plates containing 3 mL of DMEM culture medium per well, supplemented with 10% fetal bovine serum (FBS). The cultures were incubated for 24 h at 37 °C in a 5% CO_2_ atmosphere. After this period, the cells were treated as follows:

- Unstressed control: treated only with DMEM culture medium for 24 h.
- Stressed treatment: treated with 25 and 50 μg/mL of DcMt for 24 h, followed by exposure to 2 mM H_2_O_2_ for 2, 3, and 4 h.

After the incubation, the culture medium was removed, and the cells were washed with PBS and resuspended in the culture medium. The content of the culture plate wells was collected and centrifuged (2000 rpm, 10 min, at 4 °C). The pellets were resuspended in 3 mL of lysis buffer (50 mM potassium phosphate buffer, pH 7.0, 0.25% Triton X-100, 1 mM EDTA) and homogenized using an Ultra-Turrax^®^ (IKA, Staufen, Germany), (5 s, 2×). The samples were aliquoted and stored at −80 °C until used for enzymatic activity assessment. Catalase activity was evaluated following the protocol described by Aebi [51], measuring the rate of hydrogen peroxide (H_2_O_2_) decomposition.

### 4.8. Scratch Assay

This assay assessed the stimulating effect of the DcMt extract on macrophage migration, as demonstrated [52], at concentrations of 25 and 50 µg/mL. Macrophages were inoculated (2 × 10^5^ cells/well) into 24-well plates containing DMEM as the culture medium supplemented with 10% FBS. The cultures were incubated overnight at 37 °C in a 5% CO_2_ atmosphere to allow cells to adhere in a monolayer. After the incubation period, a linear wound was created in the monolayer using the tip of a sterile 200 μL plastic pipette, with the wound width set to 550 ± 50 μm. Cellular debris was removed by washing with phosphate-buffered saline (PBS). Immediately afterward, the cells were treated with DcMt at 25 and 50 µg/mL. The control received DMEM supplemented with 10% FBS, and the negative controls received DMEM supplemented with 10% FBS and DMSO at 0.4%. To prevent cell proliferation, mitomycin C (10 μg/mL) was added to the culture medium, ensuring that only cell migration was evaluated. The cells were incubated for 12 h at 37 °C in a 5% CO_2_ atmosphere. Images of the scratch area were captured immediately after the wound (time 0) and after 12 h of incubation using a camera attached to the microscope (10× magnification). The photos were analyzed using ImageJ, version 1.42q (National Institutes of Health, Bethesda, MD, USA), to determine the wound width and the rate of cell migration. The percentage of recovery area was calculated for each photograph and compared with the control group to elucidate the wound-closure capacity of the plant extracts. The experiments were performed in triplicate.

### 4.9. mRNA Extraction and Gene Expression Analysis by Real-Time PCR

The gene expression of pro- and anti-inflammatory cytokines was determined through quantitative reverse transcription real-time PCR (RT-qPCR). RAW 264.7 macrophages (2.5 × 10^5^ cells/well) were seeded in six-well plates containing DMEM supplemented with 10% fetal bovine serum (FBS) and incubated for 24 h at 37 °C in a humidified atmosphere containing 5% CO_2_. Subsequently, the cells were treated with the test extract at the indicated concentrations and incubated under the same conditions for an additional 24 h. Dexamethasone was used as a positive control. After treatment, the cells were stimulated with LPS (10 μg/mL) for 4 h. Total RNA was extracted using TRI Reagent^®^ (Sigma-Aldrich, St. Louis, MO, USA) according to the manufacturer’s instructions. The concentration and purity of the extracted RNA were assessed using a μDrop Duo Plate in a Multiskan SkyHigh spectrophotometer (Thermo Fisher Scientific, Waltham, MA, USA). Subsequently, 1000 ng of total RNA was reverse-transcribed into cDNA using the High-Capacity cDNA Reverse Transcription Kit (Thermo Fisher Scientific, Waltham, MA, USA), following the manufacturer’s instructions. The mRNA expression levels of IL-2, IL-6, IL-1β, COX-2, and IL-10 were evaluated using PowerTrack^TM^ SYBR^TM^ Green Master Mix (Thermo Fisher Scientific, Waltham, MA, USA) in a QuantStudio^TM^ 3 Real-Time PCR System (Thermo Fisher Scientific, Waltham, MA, USA). The qPCR reactions were performed in a final volume of 10 μL containing 5 μL of SYBR Green Master Mix, 1 μL of forward/reverse primer mixture (10 μM), 1 μL of cDNA, and 3 μL of RNase-free water. All reactions were performed in triplicate. The amplification protocol consisted of an initial denaturation step at 95 °C for 10 min, followed by 40 cycles at 95 °C for 30 s and 60 °C for 1 min. A melting curve analysis was performed at the end of each run to confirm amplification specificity. Gene expression was quantified according to the cycle threshold (Ct) values. β-Actin was used as the reference gene, and relative gene expression was calculated using the standard curve method.

### 4.10. Thin Layer Chromatography (TLC) Analysis

The HxMt and DcMt extracts were subjected to phytochemical prospecting in TLC aluminum plates (with 254 nm fluorescent indicator, Macherey-Nagel, Düren, Germany), in search of the presence of the classes of natural products (phenolics, flavonoids, coumarins, anthraquinone, essential oils, triterpenes/steroids, saponins, and alkaloids). Mobile phases and visualization spray reagents were used according to Wagner and Bladt [53] and compared with reference compounds. For fractions obtained from the fractionation of DcMt, the flavonoids were monitored by spraying with either NP/PEG (diphenylaminoborate/polyethyleneglycol).

### 4.11. Isolation of Compounds

DcMt (10.0 g) was subjected to liquid chromatography (silica gel-60, 0.063–0.2 mm, 70–230 mesh ASTM). The gradient elution was performed using a binary mixture with increasing polarities of hexane, dichloromethane, ethyl acetate, and ethanol. A total of 553 fractions of 50 mL each were collected and monitored by thin-layer chromatography (TLC) on 60 GF plates, yielding 30 groups of fractions (G-1 to G-30). Groups G-6, G-8, G-16, G-17, G-19, and G-20 were subjected to semipreparative HPLC separation. A volume of each group (ranging from 20 to 40 μL) diluted in acetonitrile (Merck^®^, Darmstadt, Germany) was injected on semipreparative HPLC Shimadzu Prominence with UV detector (LC—20 AR pump, SPD-20A detector, LabSolutions software Ver. 5.117, Shimadzu, Kyoto, Japan). The evaluated wavelengths were 254 and 264 nm. C-18 reverse phase column (Shimpack^®^—PREP-ODS H, 250 mm per 20 mm) (C-18) with particle diameter of 15 µm. The flow rate was 5 mL/min, with a run time of 30 min. The elution gradient of the mobile phase (phase A: ultrapure water and phase B: acetonitrile) increased linearly from 65% to 95% (*v*/*v*) of B in the time interval of 1–24 min. The system returns the initial conditions between 25 and 29 min [53]. This purification process led to the obtaining of four compounds (**1**–**4**).

### 4.12. Structural Identification

Spectroscopic techniques, such as nuclear magnetic resonance and mass spectrometry, were used to structurally identify the compounds. ^1^H and ^13^C nuclear magnetic resonance (NMR) spectra were acquired on a Bruker AscendTM 400 MHz spectrometer using the deuterated solvent CD_3_OD. Coupling constants (*J*) are presented in Hertz (Hz) and chemical shifts (δ), in ppm. The referencing of ^13^C and ^1^H resonances was performed using tetramethylsilane (TMS) as the internal standard. The 2D NMR data were recorded at 300 MHz (Bruker Avance^TM^ DRX 300, Bruker BioSpin, Rheinstetten, Germany) using heteronuclear single-quantum coherence (HSQC) and heteronuclear multiple-bond correlation (HMBC) experiments. LC–ESI-MS analysis was performed to determine the molecular masses of the isolated compounds and to establish the chromatographic profile of the DcMt extract in full-scan mode.

Chromatographic analysis was performed on Waters Acquity Ultra Performance LC equipment (Milford, MA, USA) coupled to PDA and TQ Detector, with RP-18 Acquity UPLC HSS column (100Å particles, 1.8 µm, 2.1 mm × 100 mm), flow rate of 0.3 mL/min, and column oven at 40 °C. Linear gradient elution (5–95% ACN from 0 to 10 min) with H_2_O (0.1% HCOOH)/ACN (0.1% HCOOH). Ionization by electrospray, with a full scan recorded in both positive and negative modes. Analysis parameters: capillary voltage: 3.5 kV; capillary temperature: 320 °C; desolvation temperature: 320 °C; collision gas: argon; cone voltage: 5 kV; ionization voltage: −4 kV; orifice voltage: −60 kV.

### 4.13. Statistical Analysis

Statistical analyses were performed using GraphPad Prism 5.0 software (GraphPad Software Inc., San Diego, CA, USA). Data were expressed as mean ± standard deviation (SD). Differences among groups were analyzed using one-way analysis of variance (ANOVA), followed by Tukey’s post hoc test. A *p*-value < 0.05 was considered statistically significant. All experiments were performed with *n* = 3 biological replicates.

## 5. Conclusions

The results of this study demonstrated that the *Maclura tinctoria* (DcMt) extract exhibits a high antioxidant potential, with a significant ability to neutralize free radicals and reduce ferric ions, as evidenced by the DPPH and FRAP assays. These effects can be attributed to the presence of prenylated isoflavones, known for their potent antioxidant activity. Additionally, in vitro assays revealed that DcMt exerts cytoprotective effects on RAW 264.7 macrophages exposed to H_2_O_2_-induced oxidative stress, with peak efficacy at 25 µg/mL. This protective effect was associated with activation of the catalase enzyme, indicating an antioxidant defense mechanism mediated by the extract’s prenylated isoflavones. Furthermore, the analysis of inflammatory markers revealed that DcMt at 25 µg/mL modulated the expression of key inflammatory cytokines, significantly increasing IL-10, an essential anti-inflammatory cytokine for the resolution of inflammation and tissue repair. The extract also increased IL-6 levels, which, in addition to its inflammatory role, are involved in tissue regeneration and activation of the adaptive immune response. The maintenance of IL-1β levels and the modulation of COX-2 expression suggest that DcMt promotes a controlled inflammatory response, preventing excessive inflammation. Therefore, the findings of this study highlight DcMt as a promising candidate for modulating oxidative stress and the inflammatory response in biological systems, with its effects largely driven by its prenylated isoflavones. However, to ensure its safe and effective therapeutic use, further studies are needed to clarify the mechanisms of action of its bioactive compounds and to establish the optimal doses for clinical applications. By integrating these biological activities, we aim to contribute to a more comprehensive understanding of how natural extracts can serve as viable alternatives for managing inflammatory diseases.

## Figures and Tables

**Table 1:** Retention times and identification of major compounds isolated from DcMt extract.

Compound	Retention Time (min)	Name
**1**	6.07	Lupiwighteone
**2**	6.35	Wighteone
**3**	6.93	6,8-Diprenylorobol
**4**	7.03	3,4′,5-trihydroxy-6-(3″-methylbut-2″-enyl)-7-methoxyflavonol

## Data Availability

The datasets used or analyzed during the current study are available from the corresponding author on reasonable request.

## Supplementary Materials

The following supporting information can be downloaded at: https://www.mdpi.com/article/10.3390/ijms27125504/s1.
