# Supplementary material for: Maclura tinctoria as a Modulator of Oxidative Stress and Inflammatory Responses
Source: Int J Mol Sci. 2026 Jun 18;27(12):5504. doi: 10.3390/ijms27125504 (PMC13299679; doi:10.3390/ijms27125504)
Supplement: Supplementary file 1 [file ijms-27-05504-s001.zip › ijms-4351091-supplementary.pdf]

SUPPLEMENTARY MATERIAL

Physico-chemical data of the prenylated flavonoids of *Maclura tinctoria*

5,7,4`-trihydroxy-8-[3``-methylbut-2``-enyl]-isoflavona (lupiwighteone) (1): yellowish amorphous compound. ESI-MS *m/z* (positive mode): 339 $[M+H]^+$. Molecular structure: $C_{20}H_{18}O_5$. RMN ^1H (400 MHz, CD_3OD): δ 7.93 (s, 1H, H-2), 7.35 (d, 2H, J = 8.6 Hz, H-2', 6'), 6.90 (d, 2H, J = 9.0 Hz, H-3', 5'), 6.31 (s, 1H, H-6), 5,24 (m, 1H, J = 1.5, 6.9 Hz, H-2"), 3.42 (d, 2H, J = 6.9 Hz, H-1"), 1.81 (s, 3H, H-5"), 1.68 (s, 3H, H-4"). RMN ^{13}C e DEPT135 (100 MHz, CD_3OD): δ 152,9 (C-2, CH); 122,1 (C-3, C); 181,4 (C-4, C); 159,6 (C-5, C); 98,8 (C-6, CH); 161,8 (C-7, C); 105,4 (C-8, C); 155,5 (C-9, C); 107,0 (C-10, C); 123,2 (C-1`, C); 130,2 (C-2` e C-6`, CH); 115,5 (C-3` e C-5`, CH); 157,09 (C-4`,C); 21,4 (C-1``, $CH_2$); 122,0 (C-2``, CH); 132,0 (C-3``), 17,6 (C-4``, CH_3); 25,6 (C-5``, CH_3).

5,7,4`-trihydroxy-6-[3``-methylbut-2``-enyl]-isoflavona **(wighteone) (2):** yellowish amorphous compound. ESI-MS *m/z* (positive mode): 339 $[M+H]^+$. Molecular structure: $C_{20}H_{18}O_5$. RMN ^1H (400 MHz, CD_3OD): δ 7.80 (s, 1H, H-2), 7.34 (d, 2H, J = 8.4 Hz, H-2', 6'), 6.90 (d, 2H, J = 8.4, Hz, H-3', 5'), 5,27 (m, 1H, H-2``), 3,36 (m, 2H, H-1``), 1.78 (s, 3H, H-5"), 1.69 (s, 3H, H-4"). RMN ^{13}C e DEPT135 (100 MHz, CD_3OD): δ 152,4 (C-2, CH); 122,2 (C-3, C); 181,0 (C-4, C); 159,2 (C-5, C); 112,0 (C-6, C); 162,1 (C-7, C); 93,2 (C-8, CH); 156,1 (C-9, C); 105,3 (C-10, C); 123,5 (C-1`, C); 130,2 (C-2` e C-6`, CH), 115,5 (C-3` e C-5`, CH), 157,0 (C-4`), 21,4 (C-1``, $CH_2$); 122,0 (C-2``, C); 132,1 (C-3``, C); 17,7 (C-4``, CH_3); 25,7 (C-5``, CH_3).

***5,7,3`,4`-tetrahydroxy-6-[3``-methylbut-2``-enyl]-8-[3```-methylbut-2``-enyl]-isoflavona*** **(6,8-diprenylorobol)** (**3**): yellowish amorphous compound. ESI-MS *m/z* (positive mode): 423 $[M+H]^+$. Molecular structure: $C_{25}H_{26}O_6$. $^1H$ RMN (400 MHz, $CD_3OD$): δ 7,75 (1H, s, H-2), 6.82 (m, 1H, H-6'), 6.67 (m, 1H, H-5'), 6,62 (m, 1H, H-1`), 5,09 (m, 2H, H-2``, H-2```), 3,32 (m, 4H, H-1``a, H-1``b; H-1```a, H-1```b); 1.69, (m, 6H, H-4``, H-4```), 1.61 (m, 6H, H-5``, H-5```); RMN ^{13}C e DEPT135 (100 MHz, CD_3OD): δ 153,4 (C-2, CH); 122,9 (C-3, C); 181,9 (C-4, C); 157,4 (C-5, C); 110,6 (C-6, C); 160.0 (C-7, C); 105,9 (C-8, C); 153,6 (C-9, C); 105,7 (C-10, C); 123,6 (C-1`, C); 121,5 (C-2`, CH); 144,2 (C-3`, C); 144,8 (C-4`, C); 115,6 (C-5`, CH); 116,5 (C-6`, CH);

21,9\22.0 (C-1`` e C-1```, CH_2); 121.4\121,6 (C-2`` e C-2```, CH), 135,7 (C-3``, C); 25,9 (C-4``, CH_3); 18.0(C-5``, CH_3); 134,5 (C-3```, CH); 26,0 (C-4```, CH_3); 18,1 (C-5```, CH_3).

3,5,4`-trihydroxy-6-[3``-methylbut-2``-enyl]-7-methoxyflavonol **(4):** yellowish amorphous compound. ESI-MS *m/z* (positive mode): 369 $[M+H]^+$. Molecular structure: $C_{21}H_{20}O_6$. RMN ^1H (400 MHz, CD_3OD): δ 11.96 (s, 1H, OH-C-5), 8.02 (d, 2H, *J* = 8.4 Hz, H-2', 6'), 6.88 (d, 2H, *J* = 8.8 Hz, H-3', 5'), 6.35 (s, 1H, H-8), 5,15 (m, 1H, H-2``), 3.74 (s, 3H, $OCH_3$), 3,32 (d, 2H, *J* = 6.6 Hz, H-1``), 1.71 (s, 3H, H-5"), 1.63 (s, 3H, H-4"). RMN ^{13}C (100 MHz, CD_3OD): δ 145.5 (C-2, C); 135.4 (C-3, C); 175.2 (C-4, C); 157.7 (C-5, C); 109.5 (C-6, C); 161.6 (C-7, C); 94.2 (C-8, CH); 154.9 (C-9, C); 103.5 (C-10, C); 123.3 (C-1`, C); 129.4 (C-2` e C-6`, CH); 114.0 (C-3` e C-5`, CH); 161.0 (C-4`, C); 21.4 (C-1``, $CH_2$); 121.1 (C-2``, CH); 135.8 (C-3``), 17.9 (C-4``), 25.9 (C-5``), 55.4 (OCH_3).

Figure S1. Full scan chromatogram of DcMt obtained by LC-DAD-ESI-MS

Figure S2. 1H NMR spectrum of compound **1** ($CDCl_3$, 400 MHz)

Figure S3. 13C NMR spectrum of compound **1** ($CDCl_3$, 100 MHz)

Figure S4. DEPT135 spectrum of compound **1** ($CDCl_3$, 100 MHz)

Figure S5. HMBC NMR spectrum of compound **1** ($CDCl_3$, 400 MHz), showing the heteronuclear correlation between H-2'' (δ5,24) e C8 (δ106,9)

Figure S6. Mass spectra of compound **1** obtained from the ESI-MS experiment in positive mode (A) and negative mode (B)

Figure S7. 1H NMR spectrum of compound **2** ($CDCl_3$, 400 MHz)

Figure S8. - 13C NMR spectrum of compound **2** ($CDCl_3$, 100 MHz)

Figure S9. - DEPT135 spectrum of compound **2** ($CDCl_3$, 100 MHz)13

Figure S10. - HMBC NMR spectrum of compound **2** ($CDCl_3$, 400 MHz), showing the heteronuclear correlation between H-2'' (δ5,27) e C6 (δ112,0); H-1'' (δ1,79) e C5 (δ159,0)

Figure S11. Mass spectra of compound **2** obtained from the ESI-MS experiment in positive mode (A) and negative mode (B)

Figure S12. 1H NMR spectrum of compound **3** ($CDCl_3$, 400 MHz)

Figure S13. 13C NMR spectrum of compound **3** ($CDCl_3$, 100 MHz)

Figure S14. DEPT135 spectrum of compound **3** ($CDCl_3$, 100 MHz)

Figure S15. HMBC NMR spectrum of compound **3** ($CDCl_3$, 400 MHz), showing the heteronuclear correlation between H-1''' (δ3,32) e C7 (δ157,3); H-1''' (δ3,32) e C9 (δ153,3)

Figure S16. HSQC NMR spectrum of compound **3** ($CDCl_3$, 100 MHz para ^{13}C e 400 MHz para ^{1}H)

Figure S17. Mass spectra of compound **3** obtained from the ESI-MS experiment in positive mode (A) and negative mode (B)

Figure S18. 1H NMR spectrum of compound **4** ($CDCl_3$, 400 MHz)

Figure S19. 13C NMR spectrum of compound **4** ($CDCl_3$, 100 MHz)

Figure S20. DEPT135 spectrum of compound **4** ($CDCl_3$, 100 MHz)

Figure S21. HMBC NMR spectrum of compound **4** ($CDCl_3$, 400 MHz),), showing the heteronuclear correlation between H-1'' (δ3,36) e C6 (δ109,5); H-1'' (δ3,36) e C2'' (δ121,15); H-1'' (δ3,36) e C6 (δ109,5); OCH2- H (δ3,74) e C7 (δ161,6); H-1'' (δ3,36) e C5 (δ157,8); H-1'' (δ3,36) e C7 (δ161,6)

Figure S22. HSQC NMR spectrum of compound **4** ($CDCl_3$, 100 MHz para ^{13}C e 400 MHz para ^{1}H), showing the heteronuclear correlation between H-1'' (δ3,36) e C-1'' (δ161,6)

Figure S23. Mass spectra of compound **4** obtained from the ESI-MS experiment in positive mode (A) and negative mode (B)

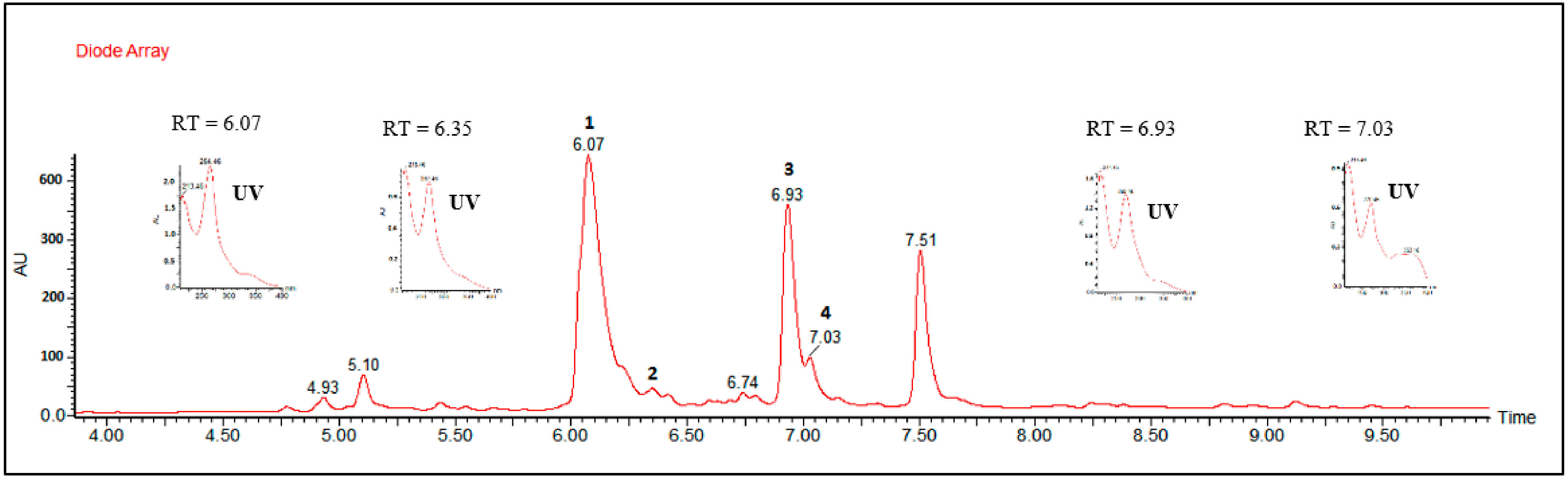

Figure S1. Full scan chromatogram of dichloromethane extract of *Maclura tinctoria* (DcMt) obtained by LC-DAD-ESI-MS: Waters Acquity Ultra Performance LC, PDA and TQ Detector, RP-18 Acquity UPLC HSS column (100Å particles, 1.8 µm, 2.1 mm × 100 mm), flow rate of 0.3 mL/min, and column oven at 40 ºC. Linear gradient elution (5–95% ACN from 0 to 10 min) with H_2O (0.1% HCOOH)/ACN (0.1% HCOOH) with UV spectra registered online for peaks corresponding to compound 1 (RT = 6.07 min), compound 2 (RT = 6.35 min), compound 3 (RT = 6.93 min), and compound 4 (RT = 7.03 min). Detection: 280 nm.

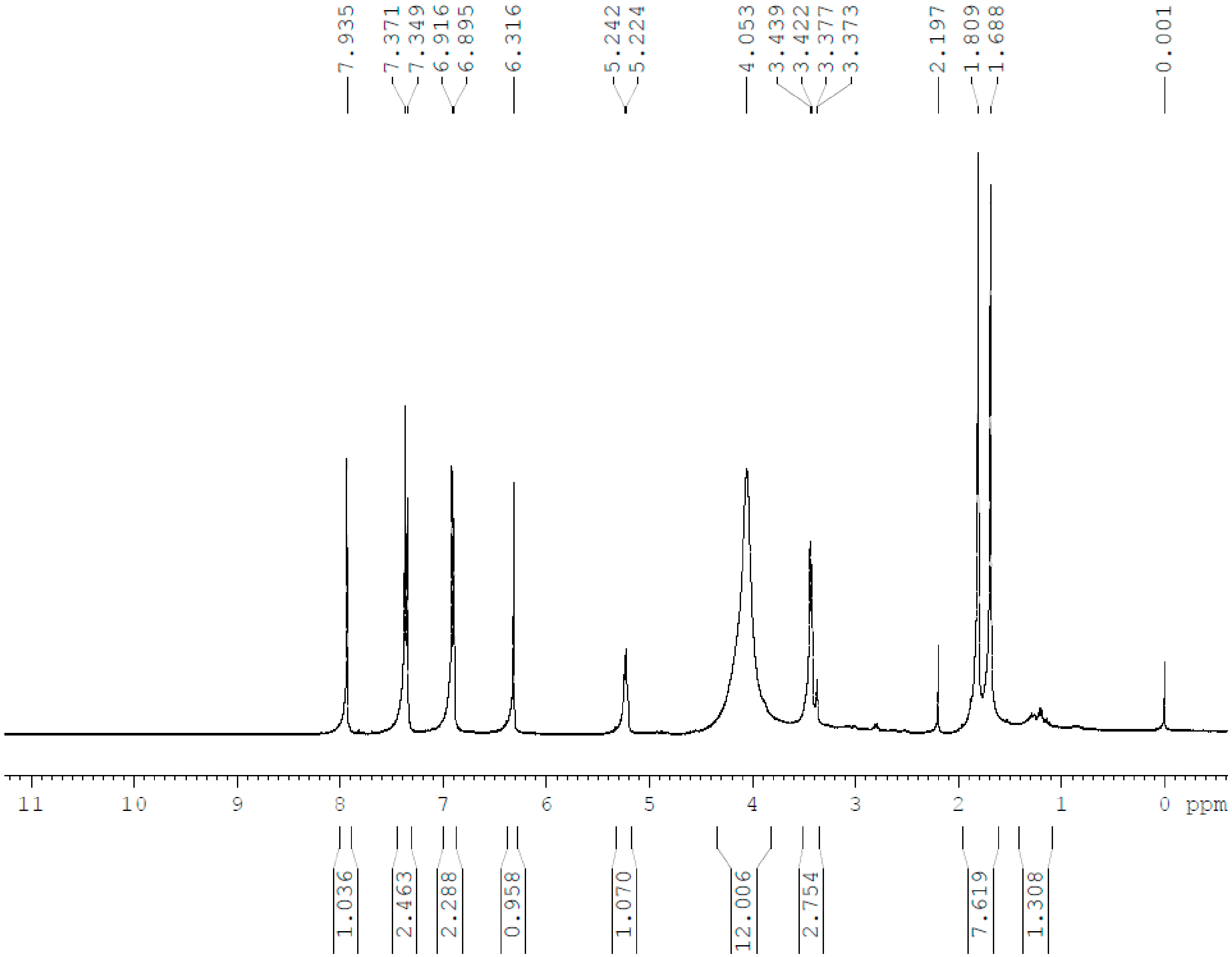

Figure S2. 1H NMR spectrum of compound **1** ($CDCl_3$, 400 MHz)

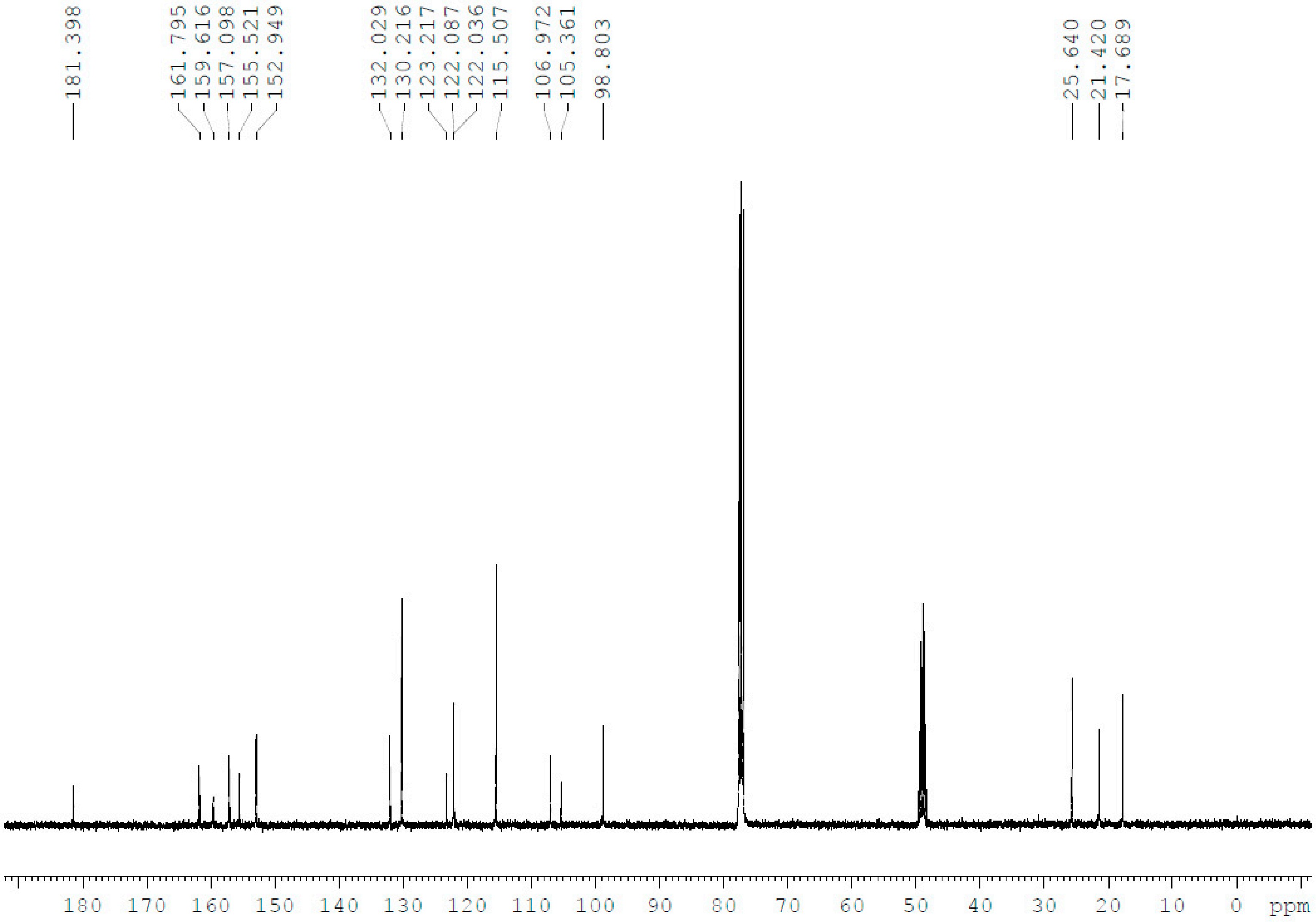

Figure S3. 13C NMR spectrum of compound **1** ($CDCl_3$, 100 MHz)

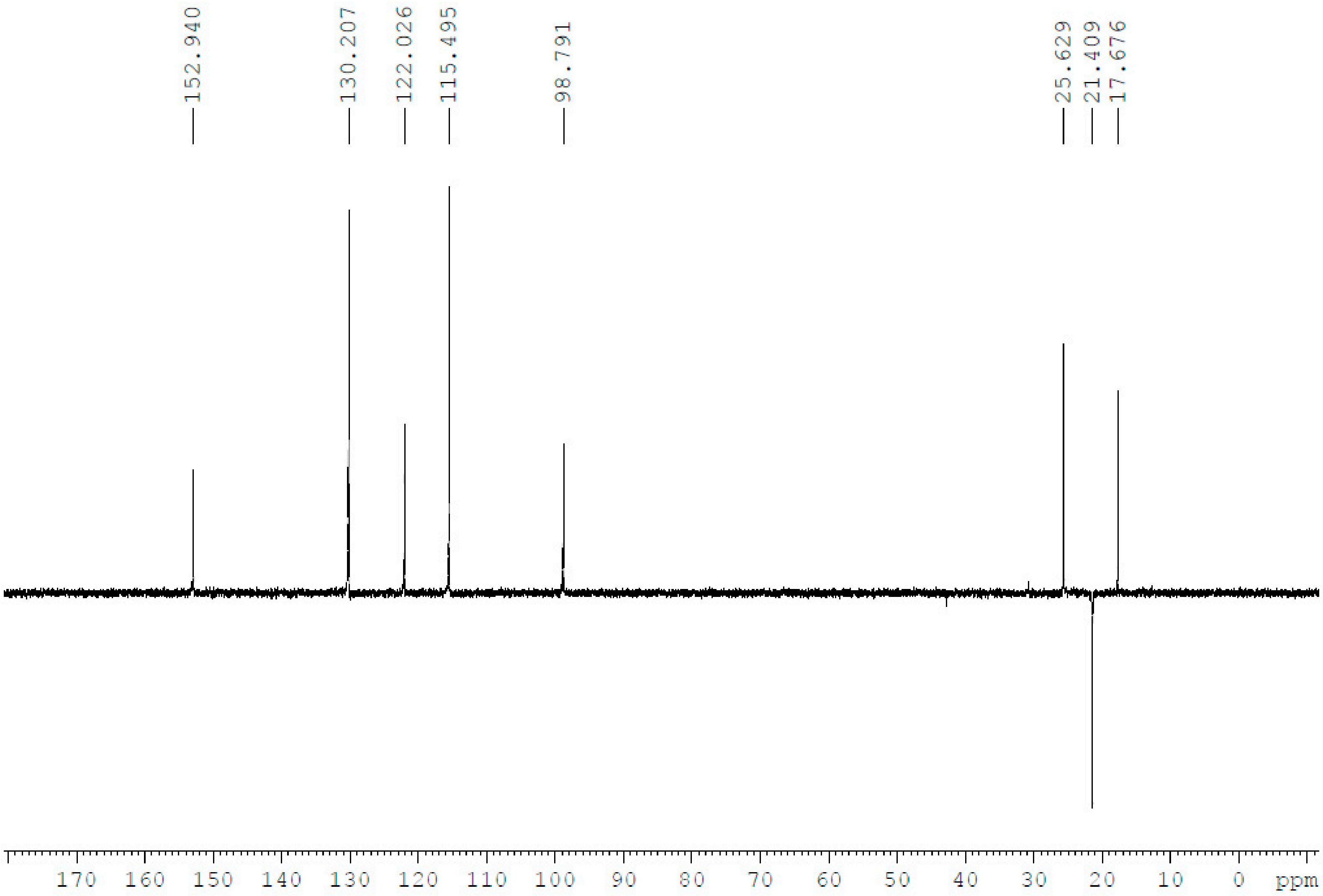

Figure S4. DEPT135 spectrum of compound **1** ($CDCl_3$, 100 MHz)

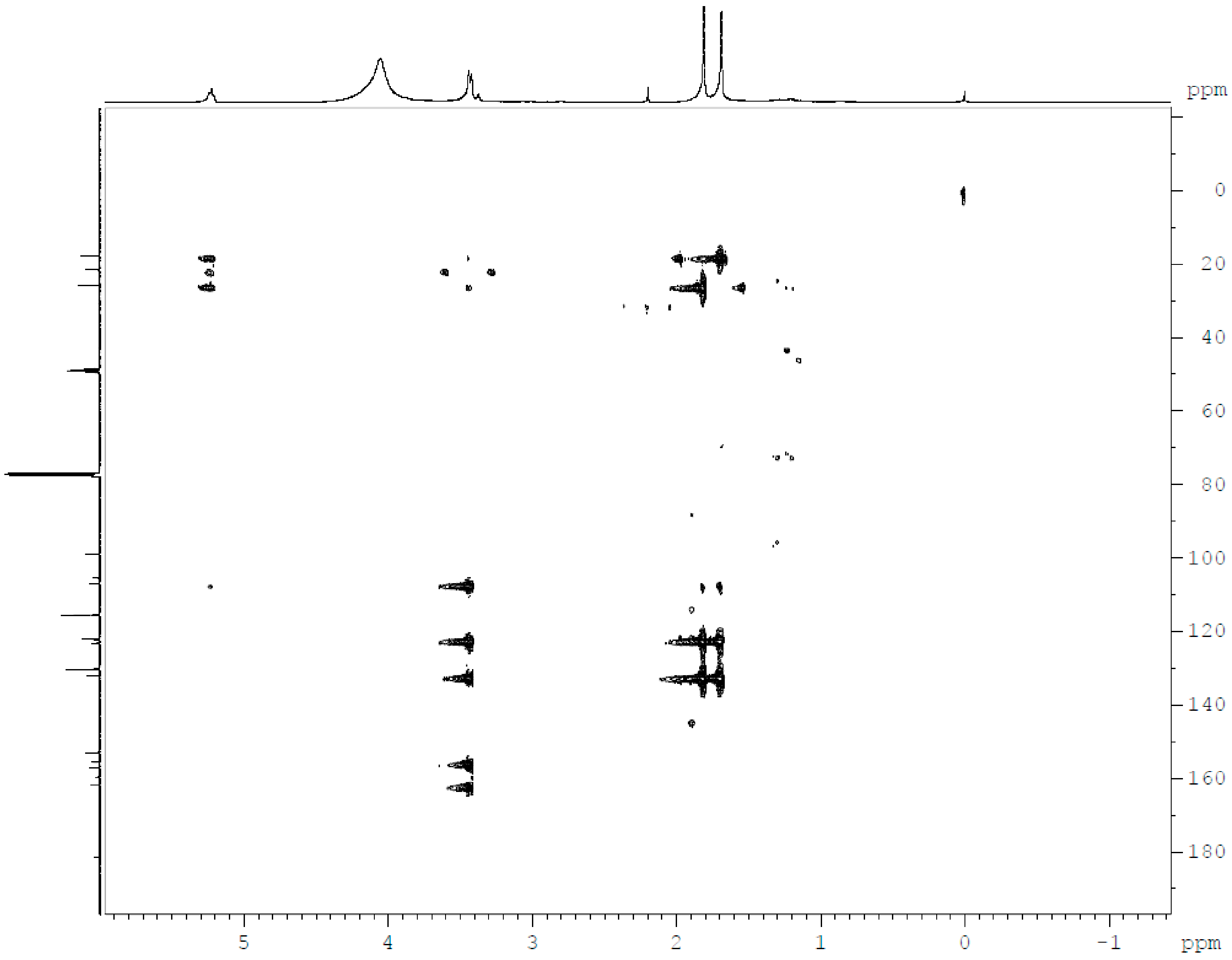

Figure S5. HMBC NMR spectrum of compound **1** ($CDCl_3$, 400 MHz), showing the heteronuclear correlation between H-2’’ (δ5,24) e C8 (δ106,9)

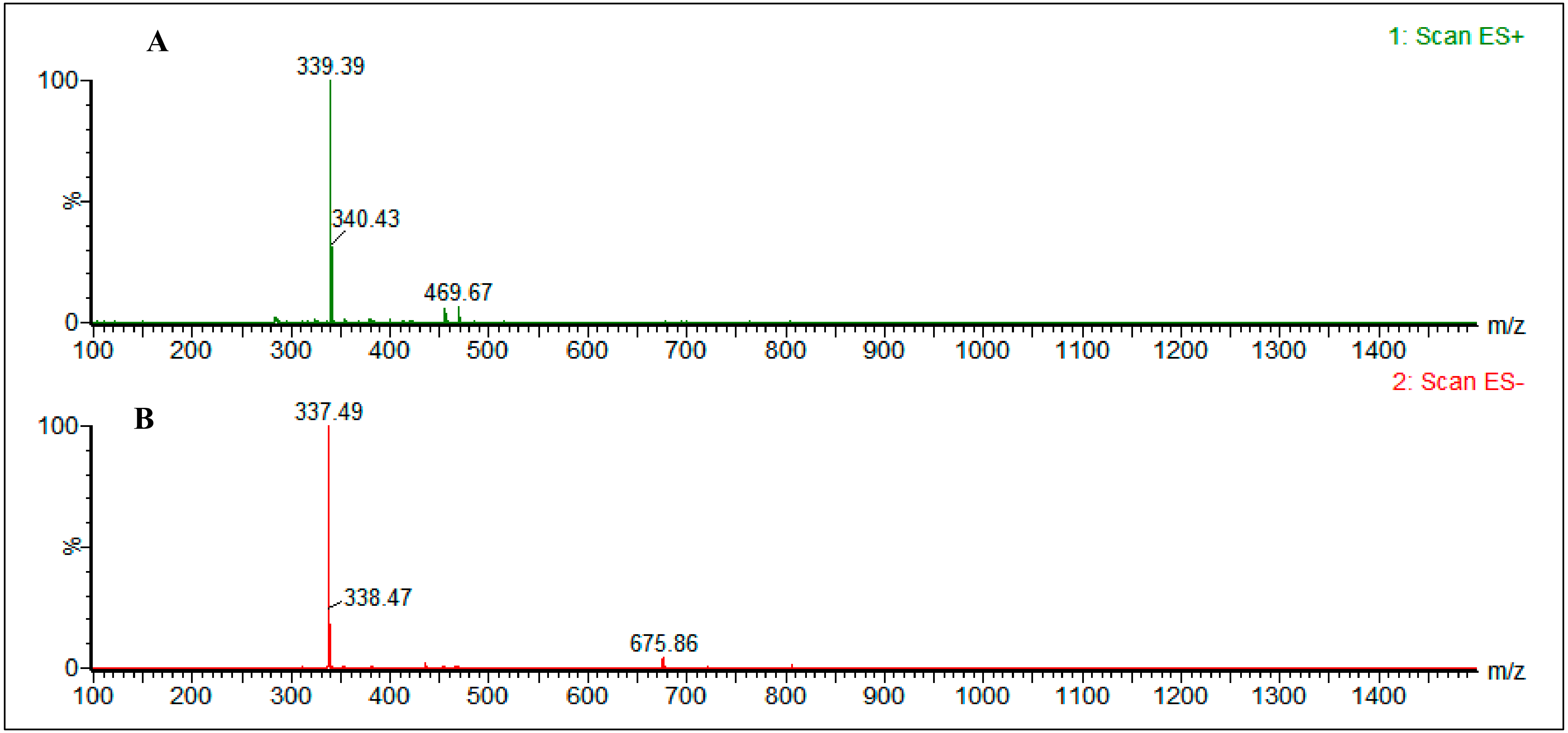

Figure S6. Mass spectra of compound **1** obtained from the ESI-MS experiment in positive mode (A) and negative mode (B)

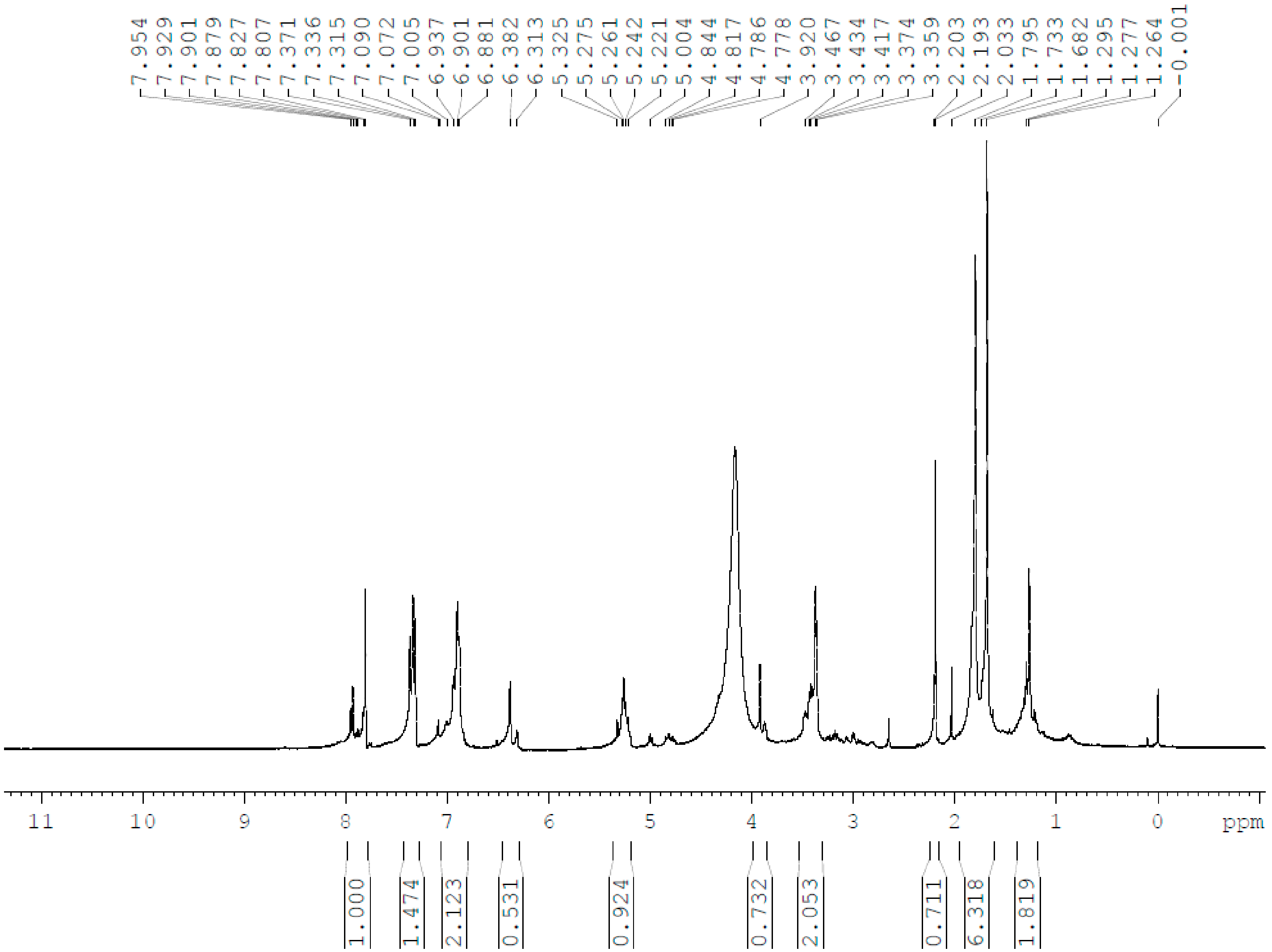

Figure S7. 1H NMR spectrum of compound **2** ($CDCl_3$, 400 MHz)

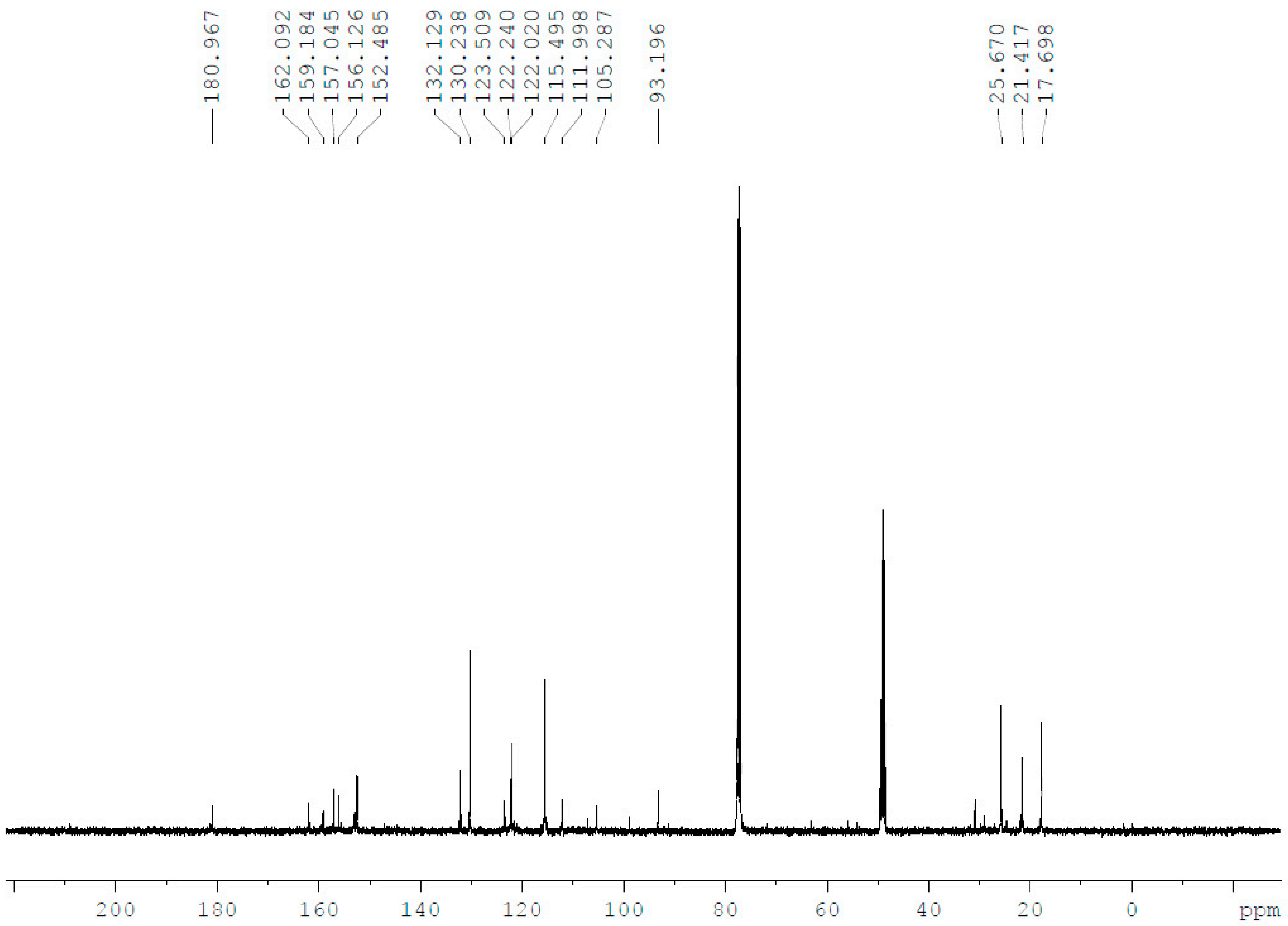

Figure S8. 13C NMR spectrum of compound **2** ($CDCl_3$, 100 MHz)

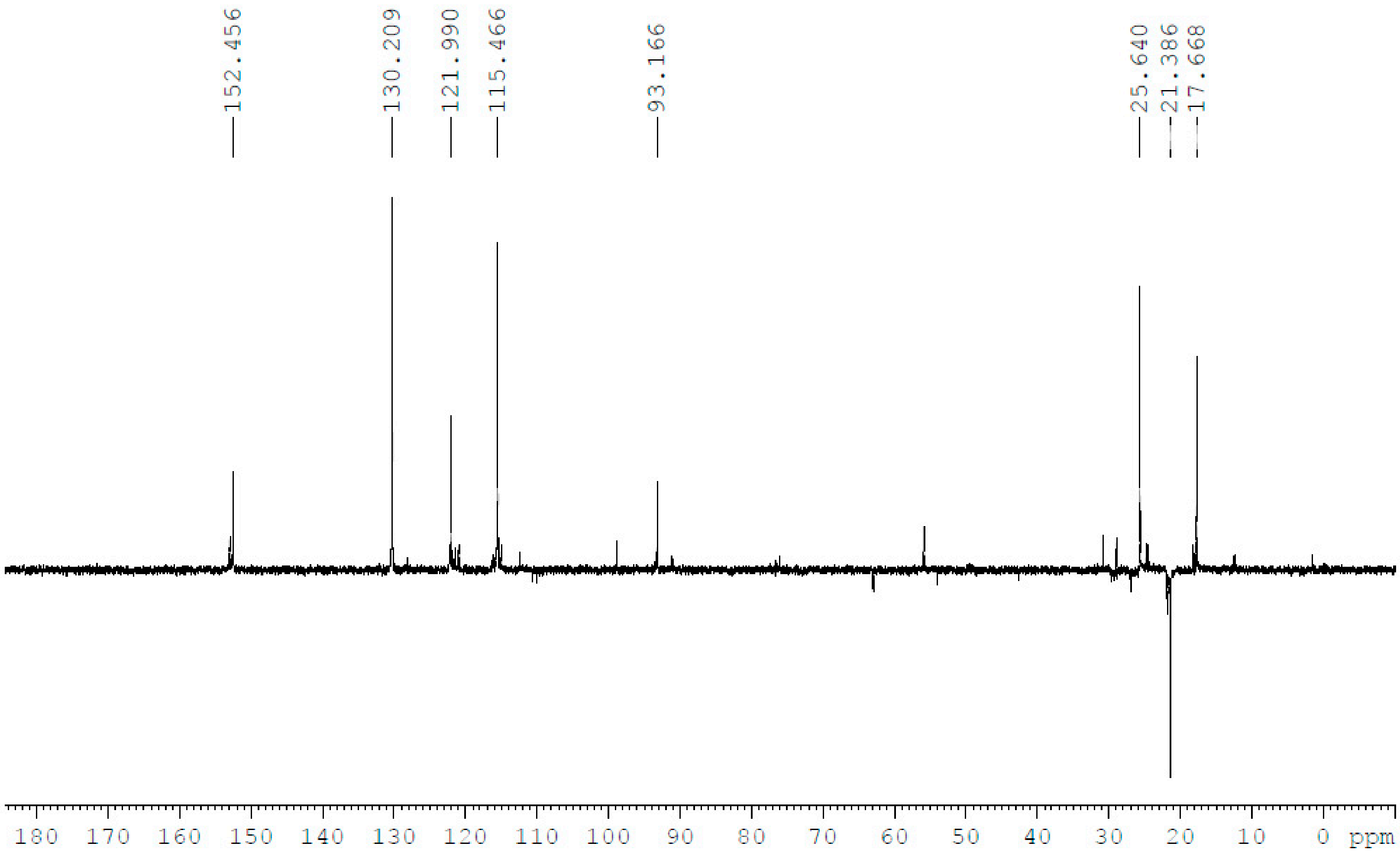

Figure S9. DEPT135 spectrum of compound **2** ($CDCl_3$, 100 MHz)

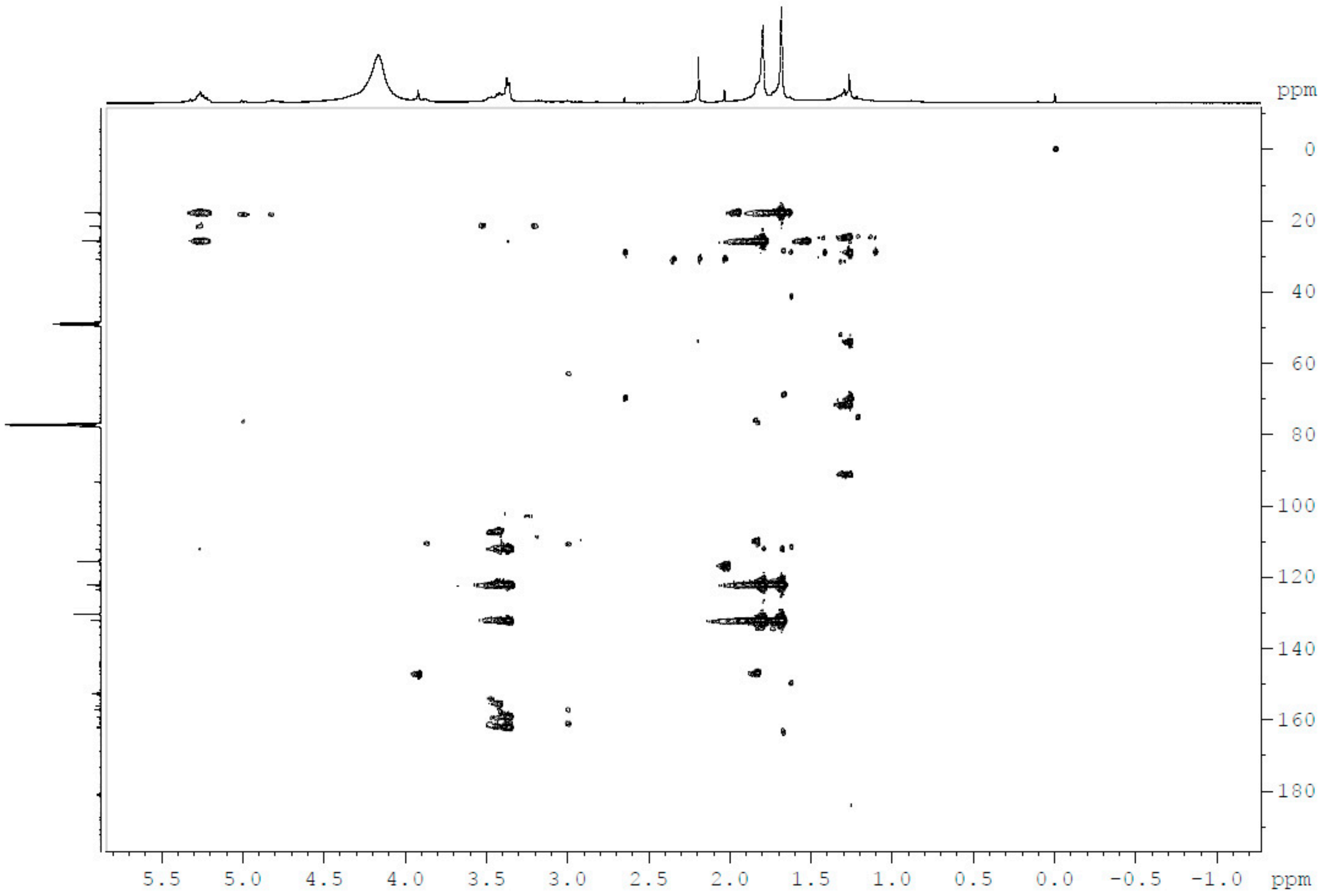

Figure S10. HMBC NMR spectrum of compound **2** ($CDCl_3$, 400 MHz), showing the heteronuclear correlation between H-2’’ (δ5,27) e C6 (δ112,0); H-1’’ (δ1,79) e C5 (δ159,0).

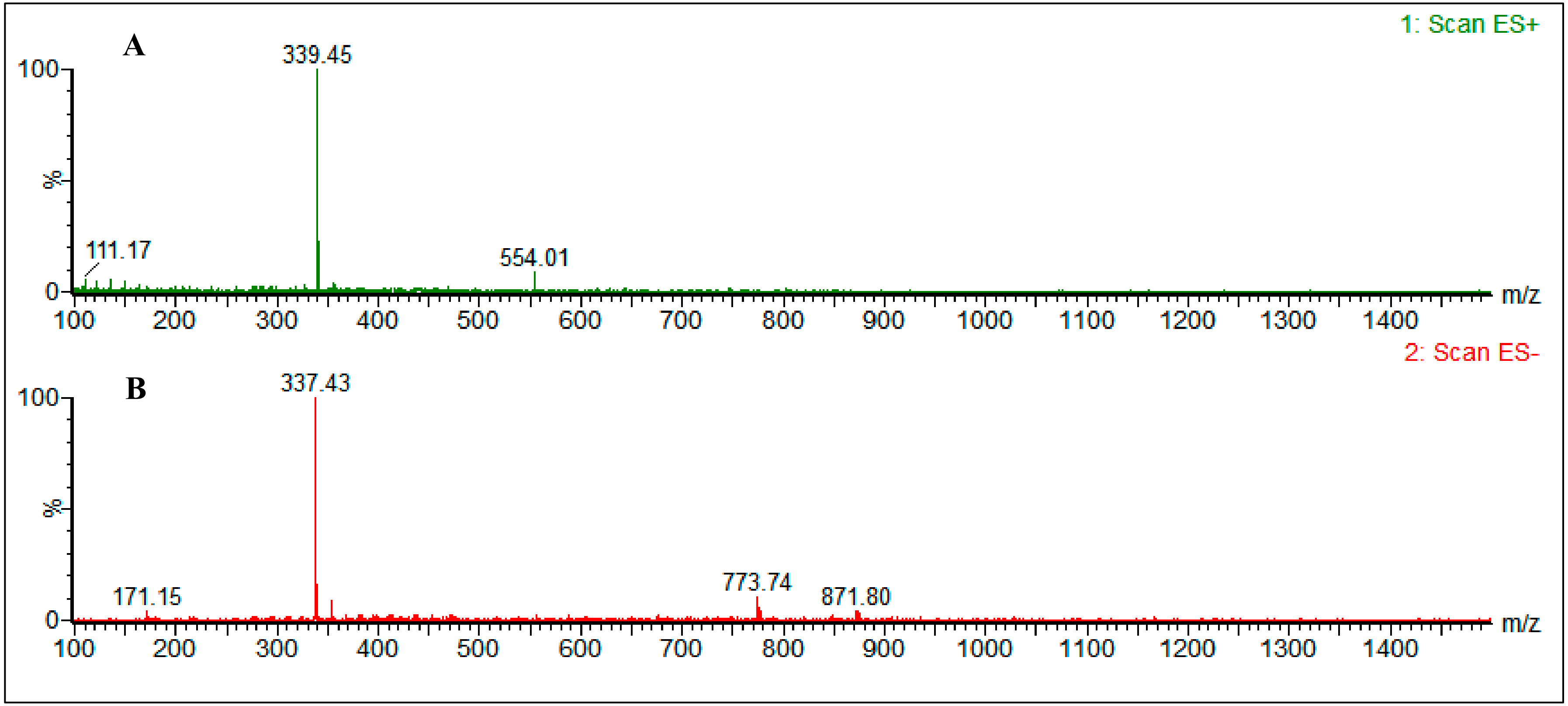

Figure S11. Mass spectra of compound **2** obtained from the ESI-MS experiment in positive mode (A) and negative mode (B)

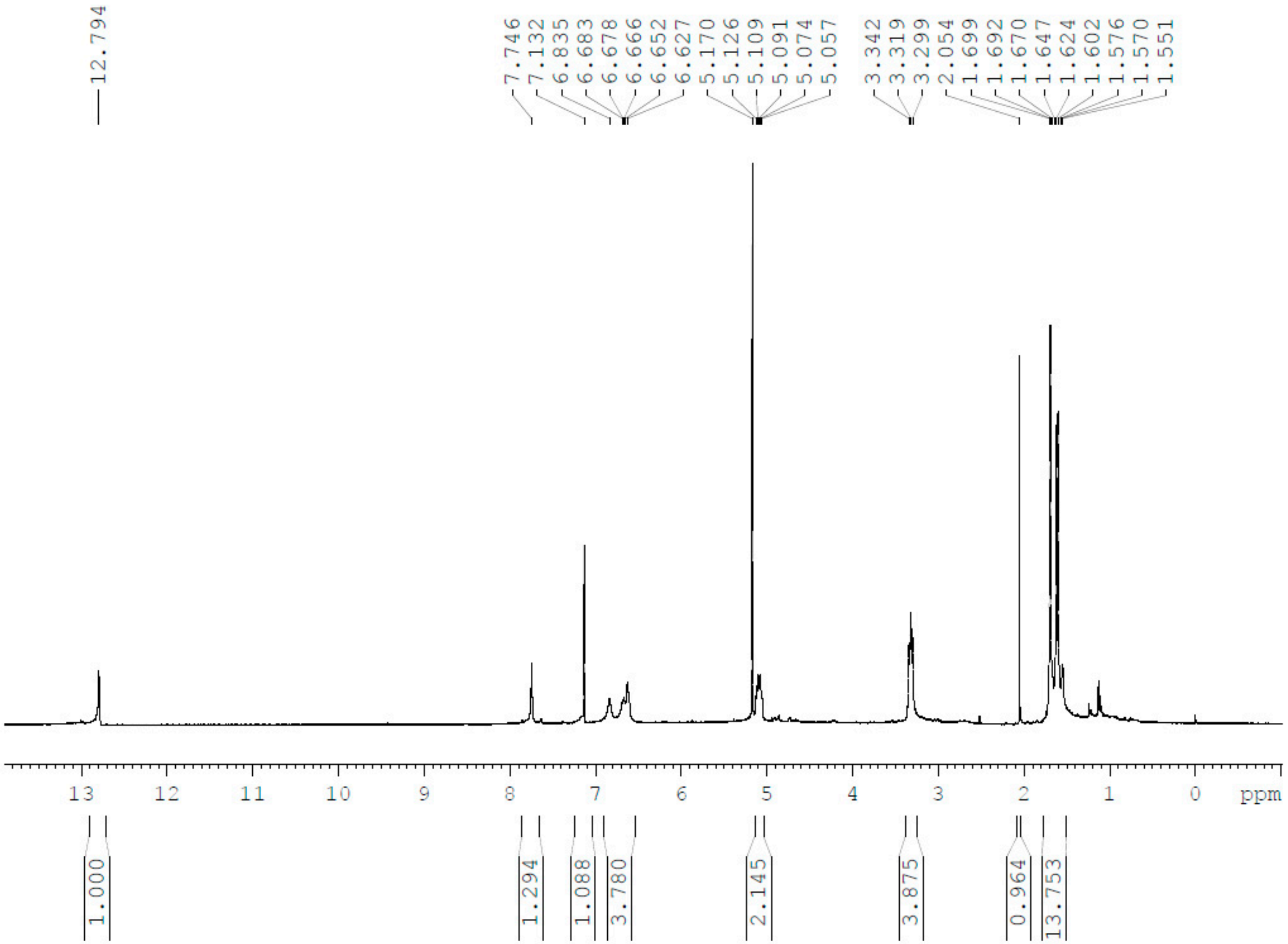

Figure S12. 1H NMR spectrum of compound **3** ($CDCl_3$, 400 MHz)

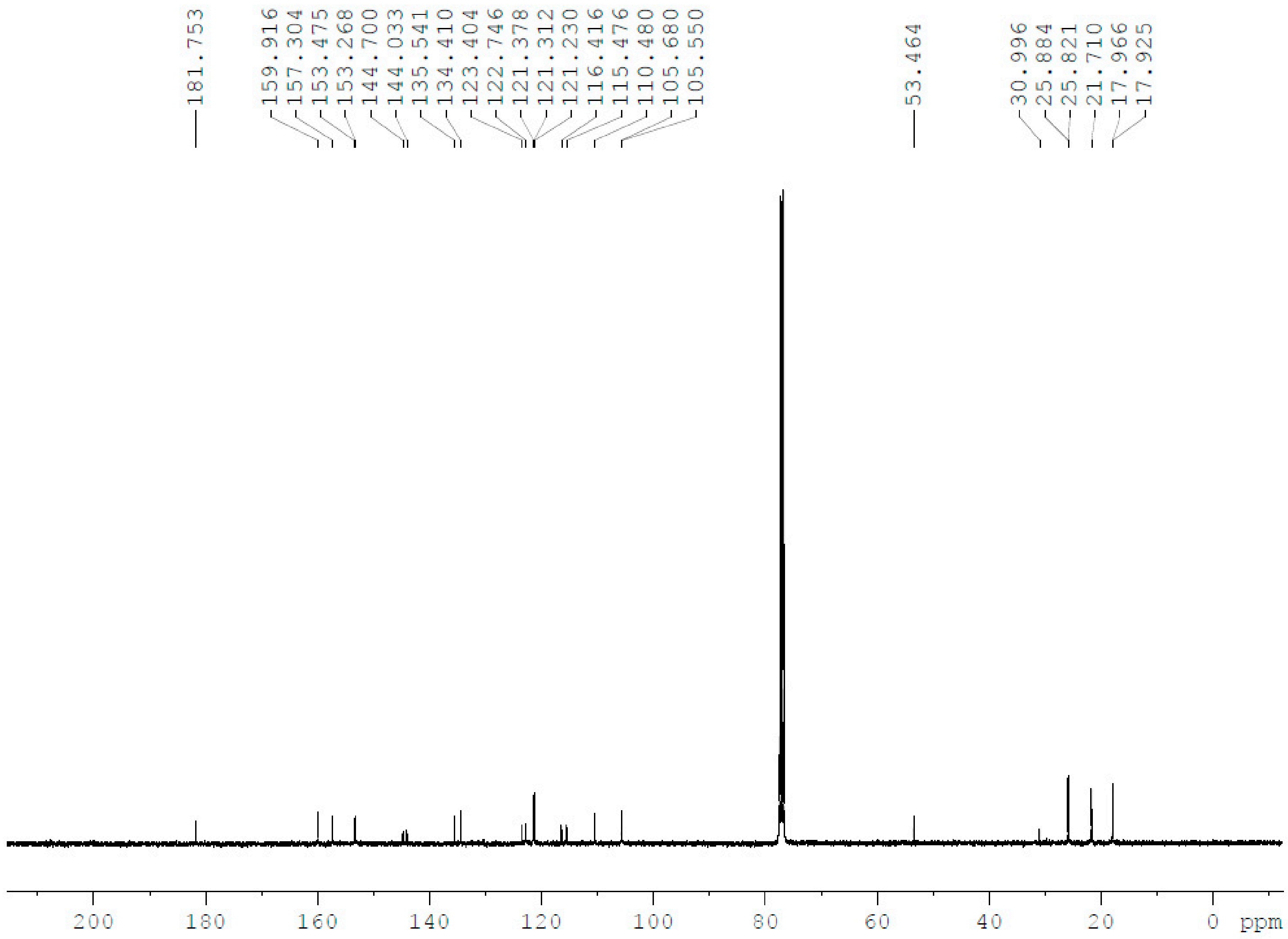

Figure S13. 13C NMR spectrum of compound **3** ($CDCl_3$, 100 MHz)

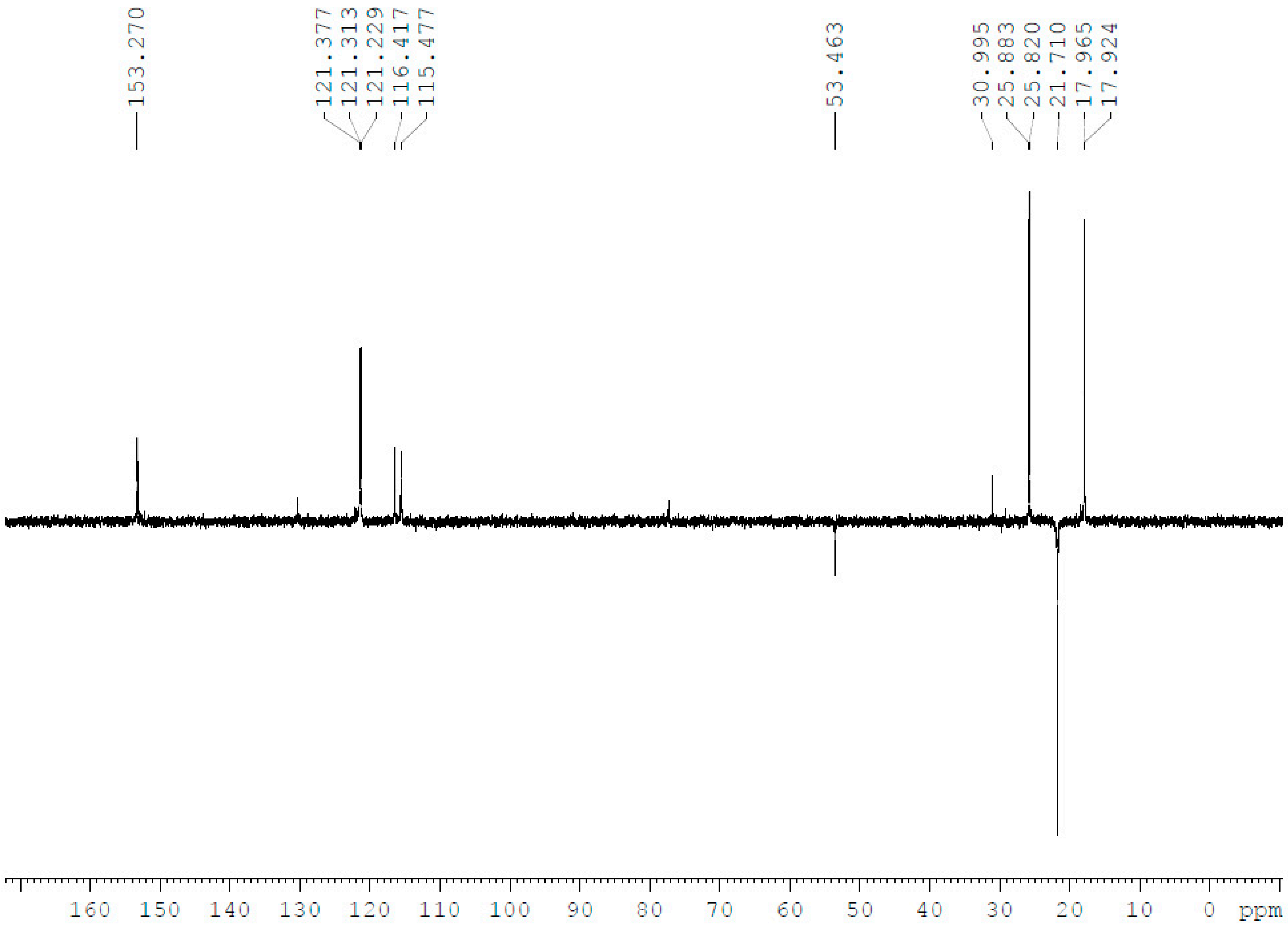

Figure S14. DEPT135 spectrum of compound **3** ($CDCl_3$, 100 MHz)

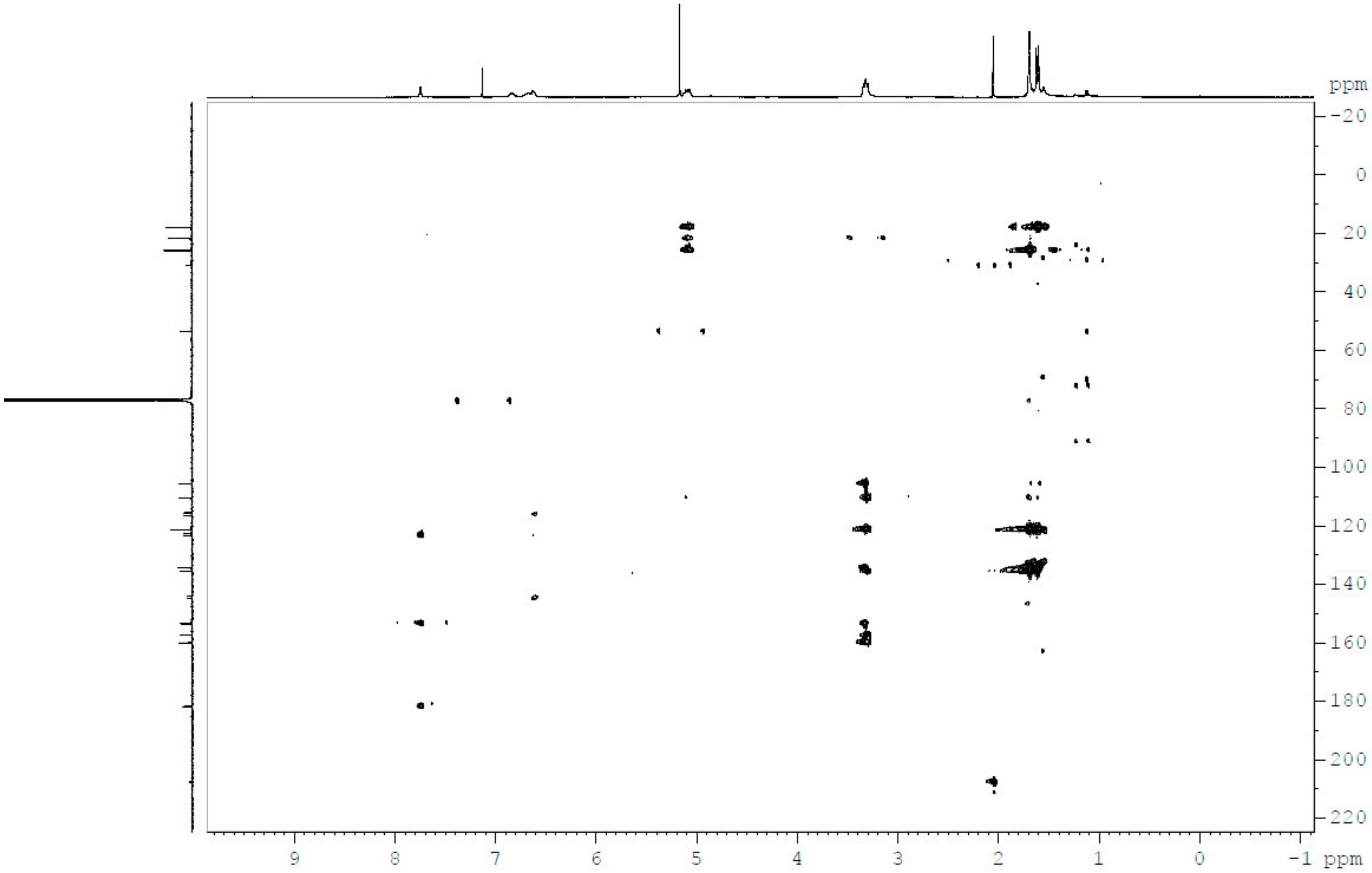

Figure S15. HMBC NMR spectrum of compound **3** ($CDCl_3$, 400 MHz), showing the heteronuclear correlation between H-1''' (δ3,32) e C7 (δ157,3); H-1''' (δ3,32) e C9 (δ153,3)

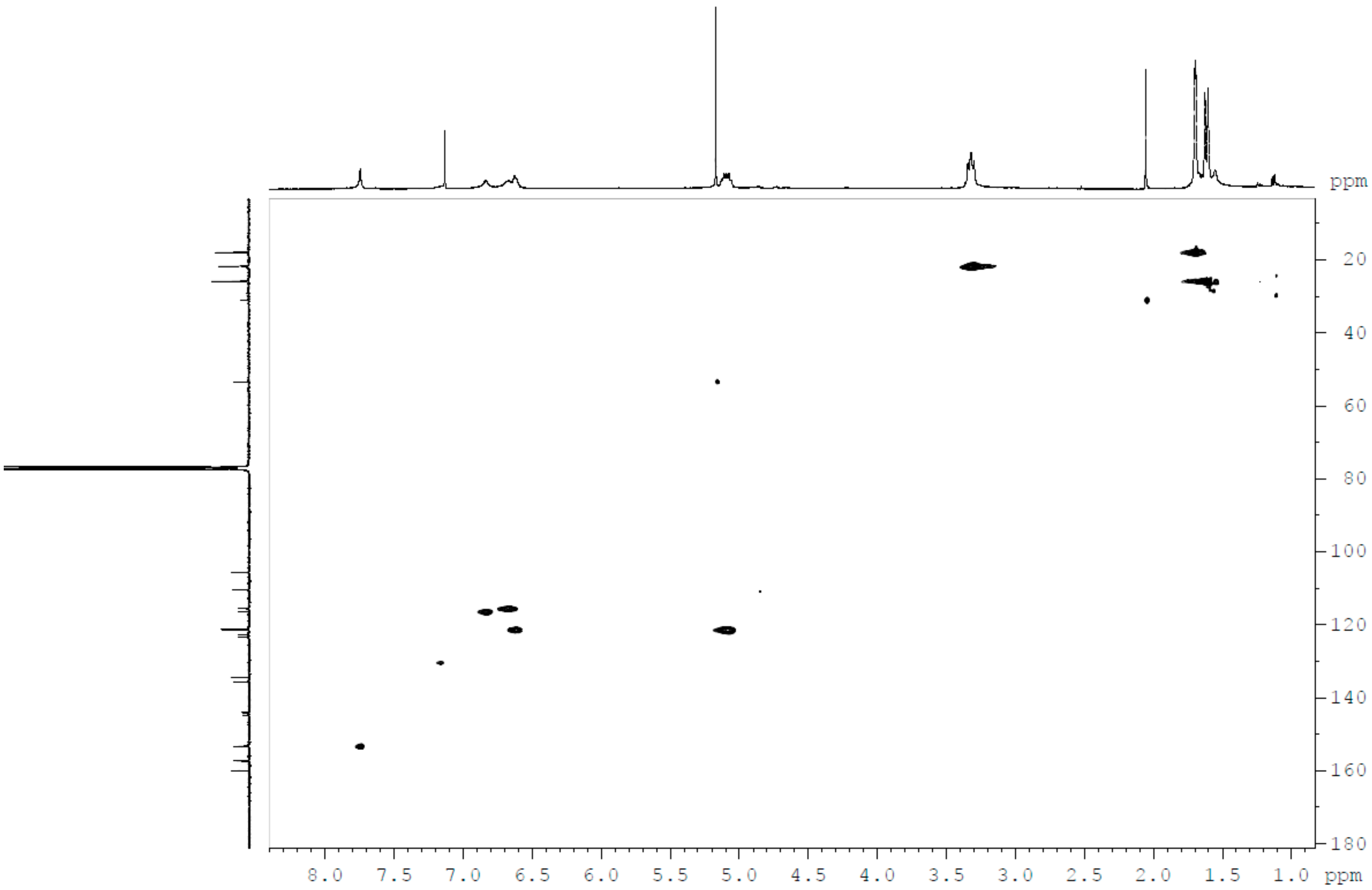

Figure S16. HSQC NMR spectrum of compound **3** ($CDCl_3$, 100 MHz para 13C e 400 MHz para 1H)

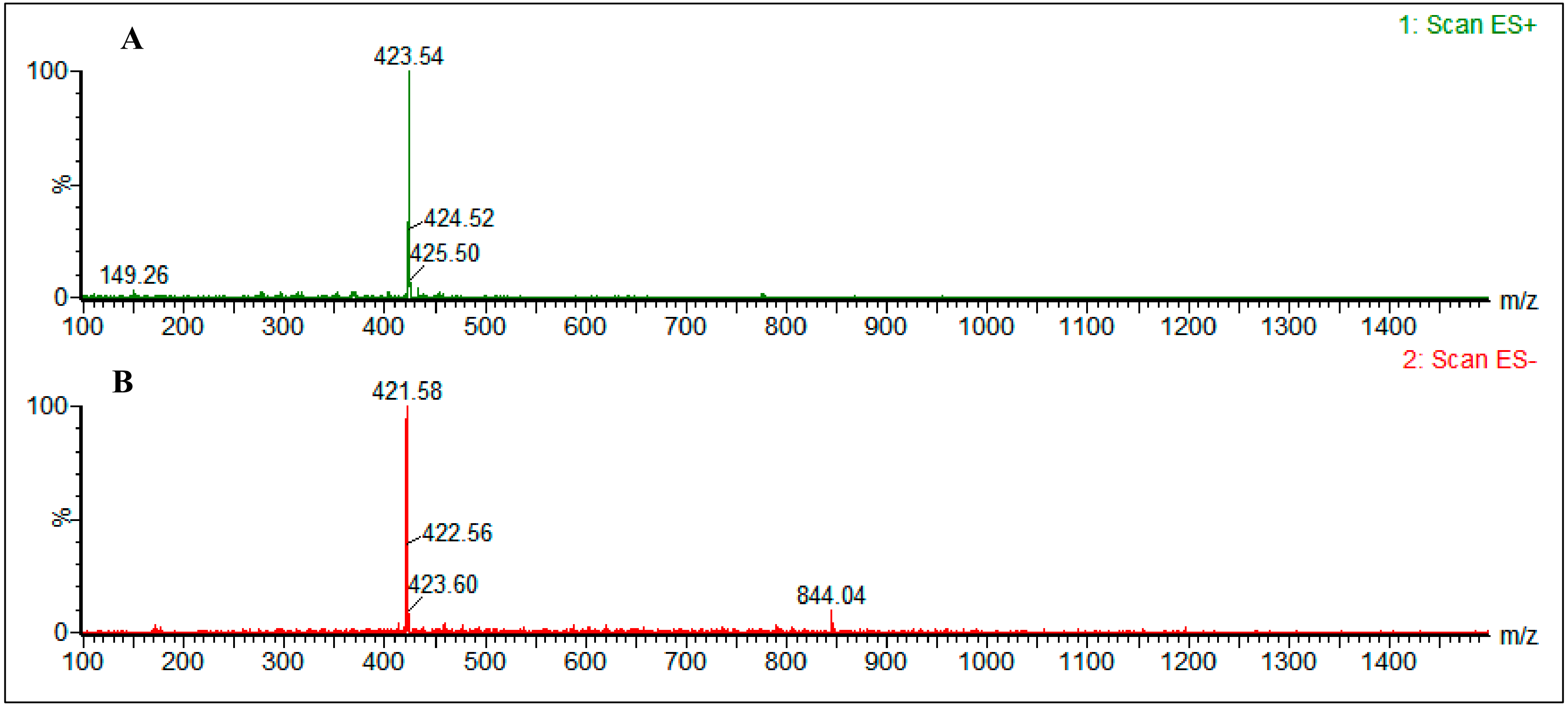

Figure S17. Mass spectra of compound **3** obtained from the ESI-MS experiment in positive mode (A) and negative mode (B)

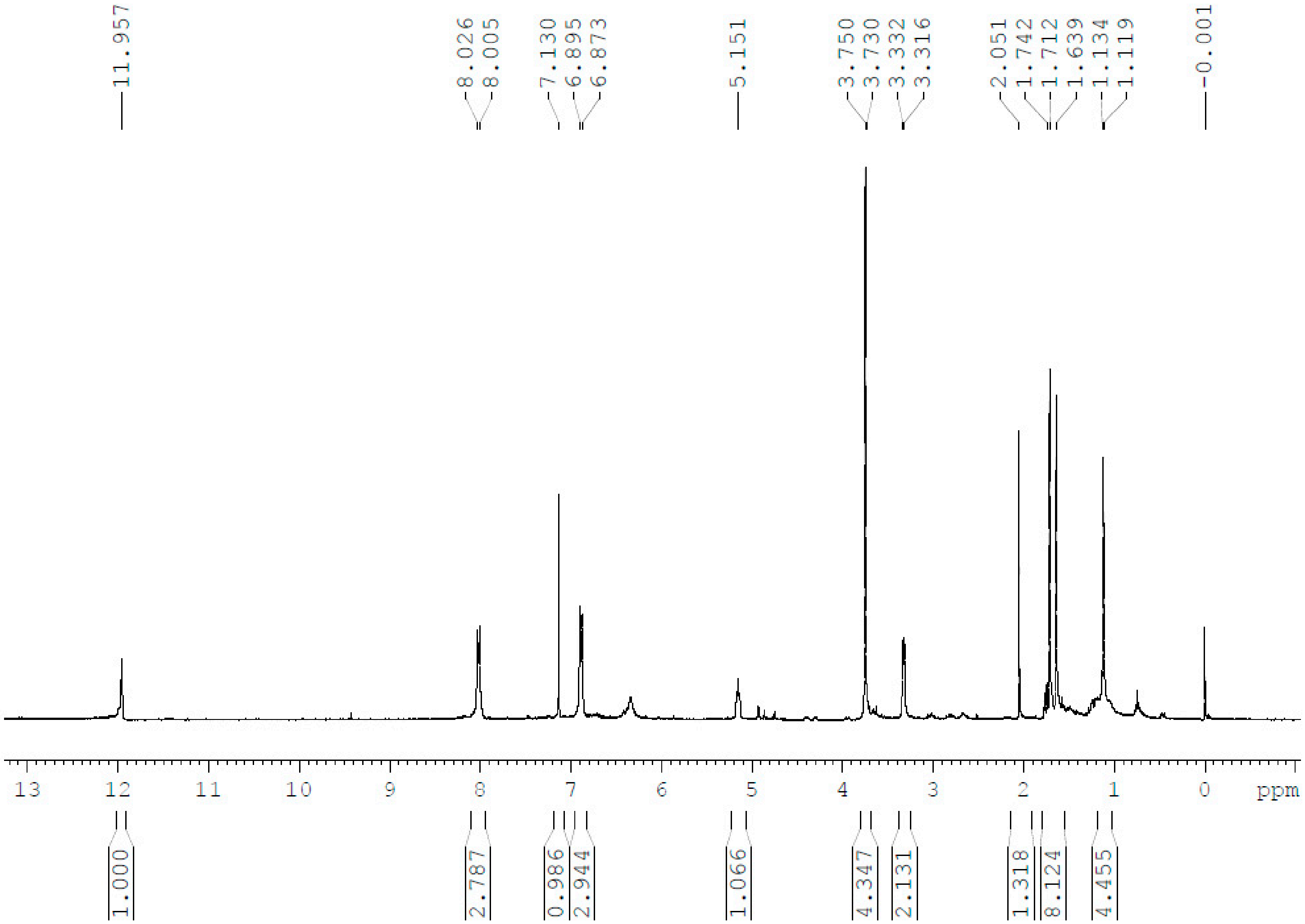

Figure S18. 1H NMR spectrum of compound **4** ($CDCl_3$, 400 MHz)

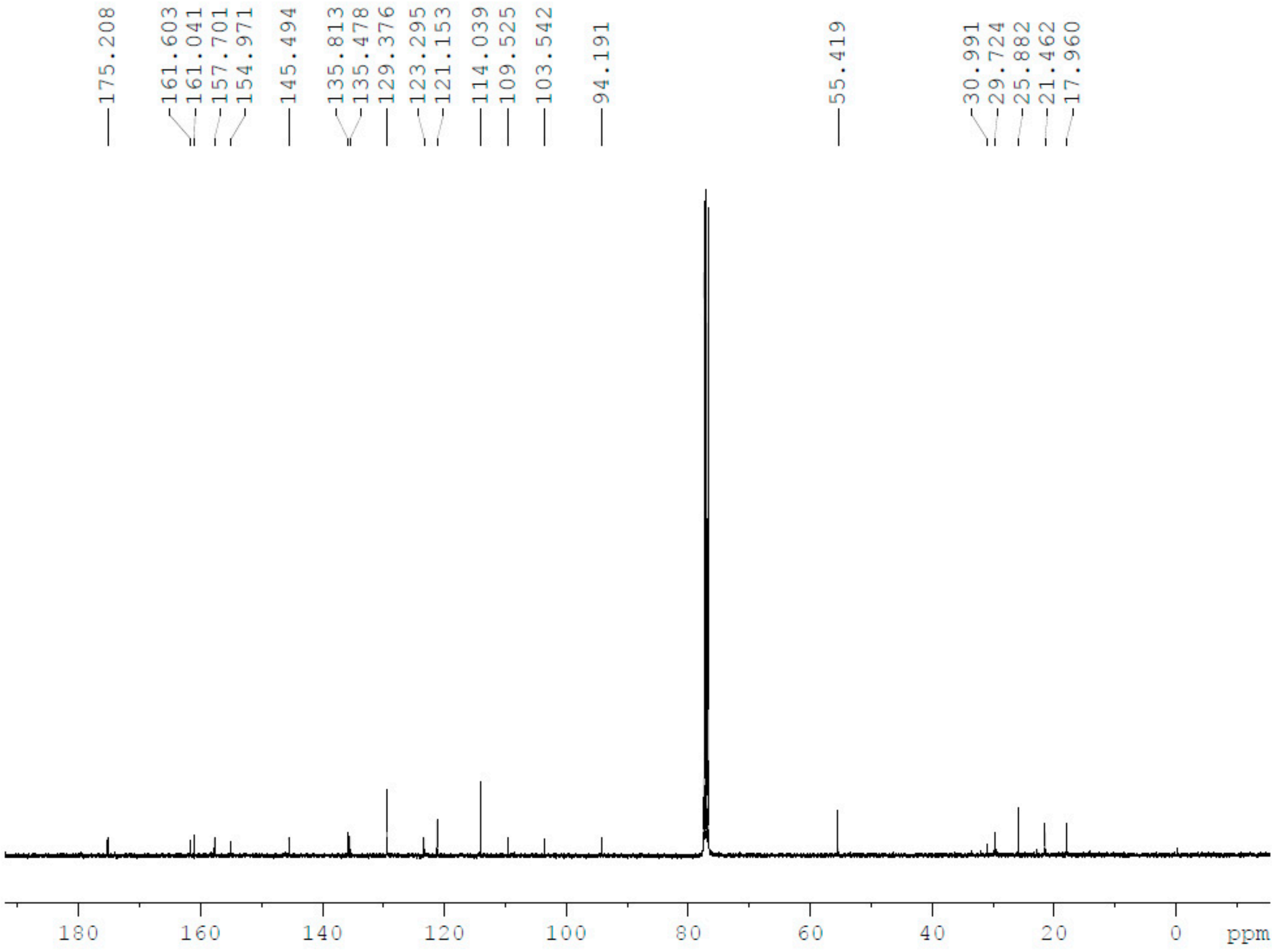

Figure S19. 13C NMR spectrum of compound **4** ($CDCl_3$, 100 MHz)

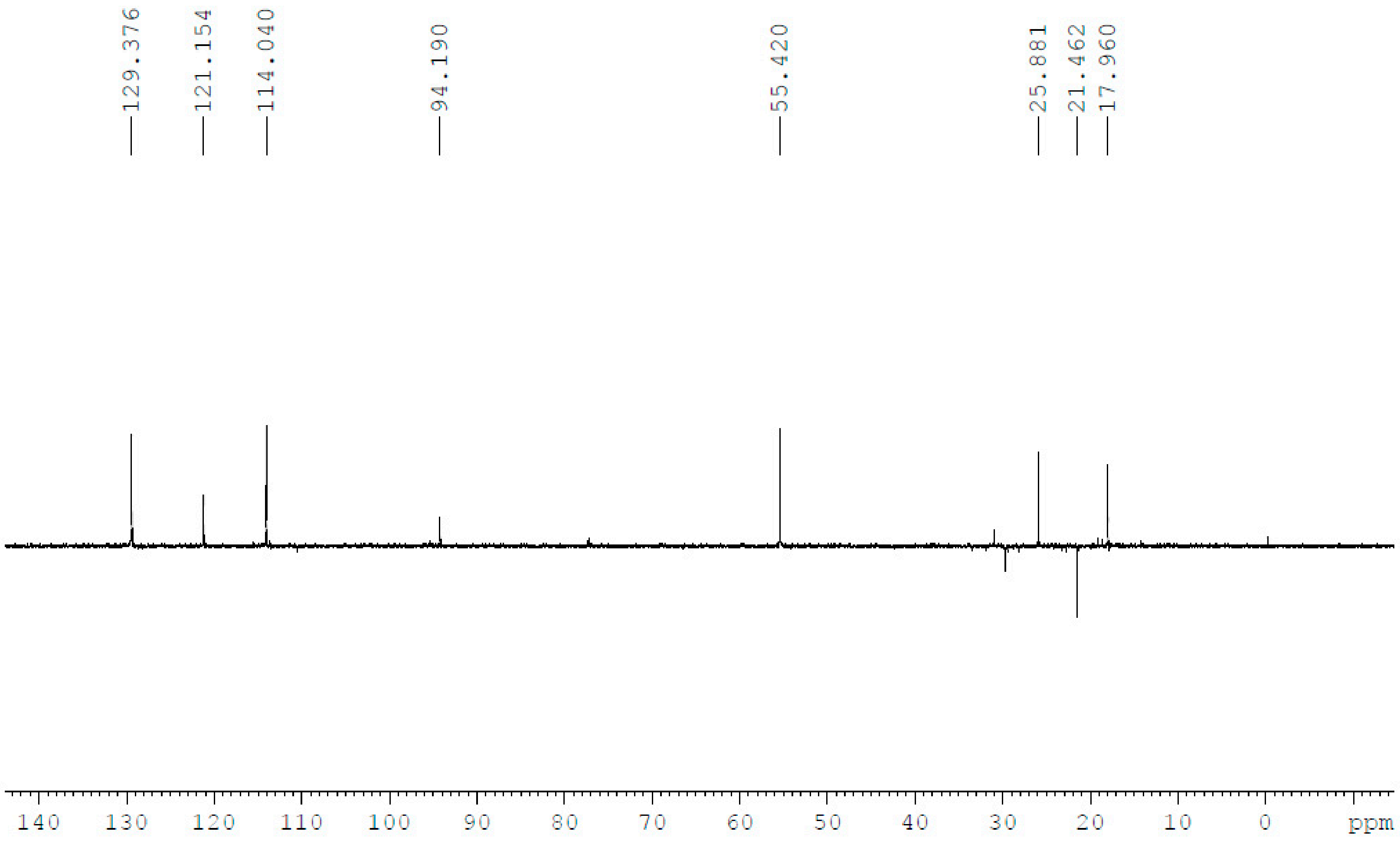

Figure S20. DEPT135 spectrum of compound **4** ($CDCl_3$, 100 MHz)

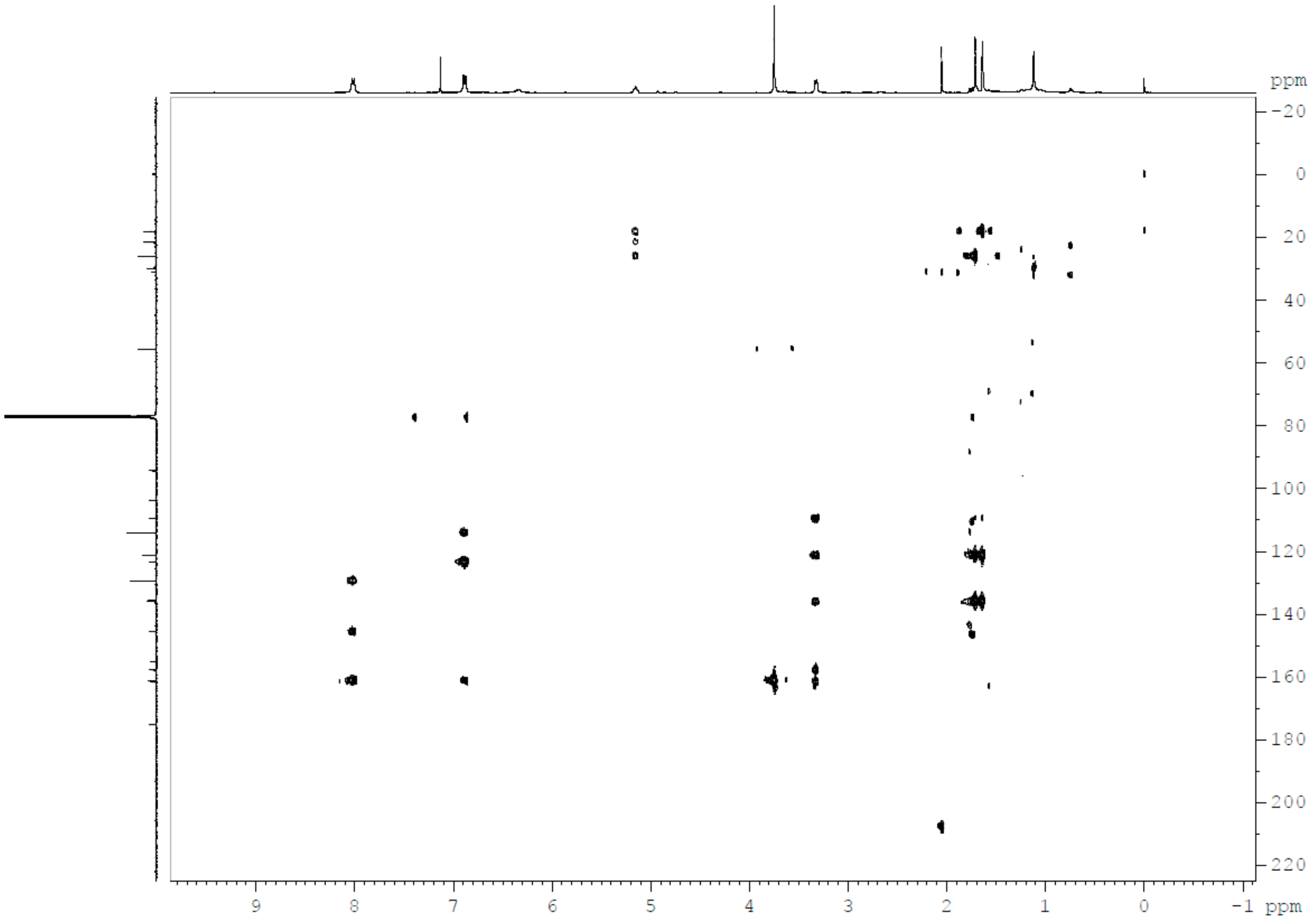

Figure S21. HMBC NMR spectrum of compound **4 (**$CDCl_3$, 400 MHz),), showing the heteronuclear correlation between H-1'' (δ3,36) e C6 (δ109,5); H-1'' (δ3,36) e C2'' (δ121,15); H-1'' (δ3,36) e C6 (δ109,5); OCH2- H (δ3,74) e C7 (δ161,6); H-1'' (δ3,36) e C5 (δ157,8); H-1'' (δ3,36) e C7 (δ161,6)

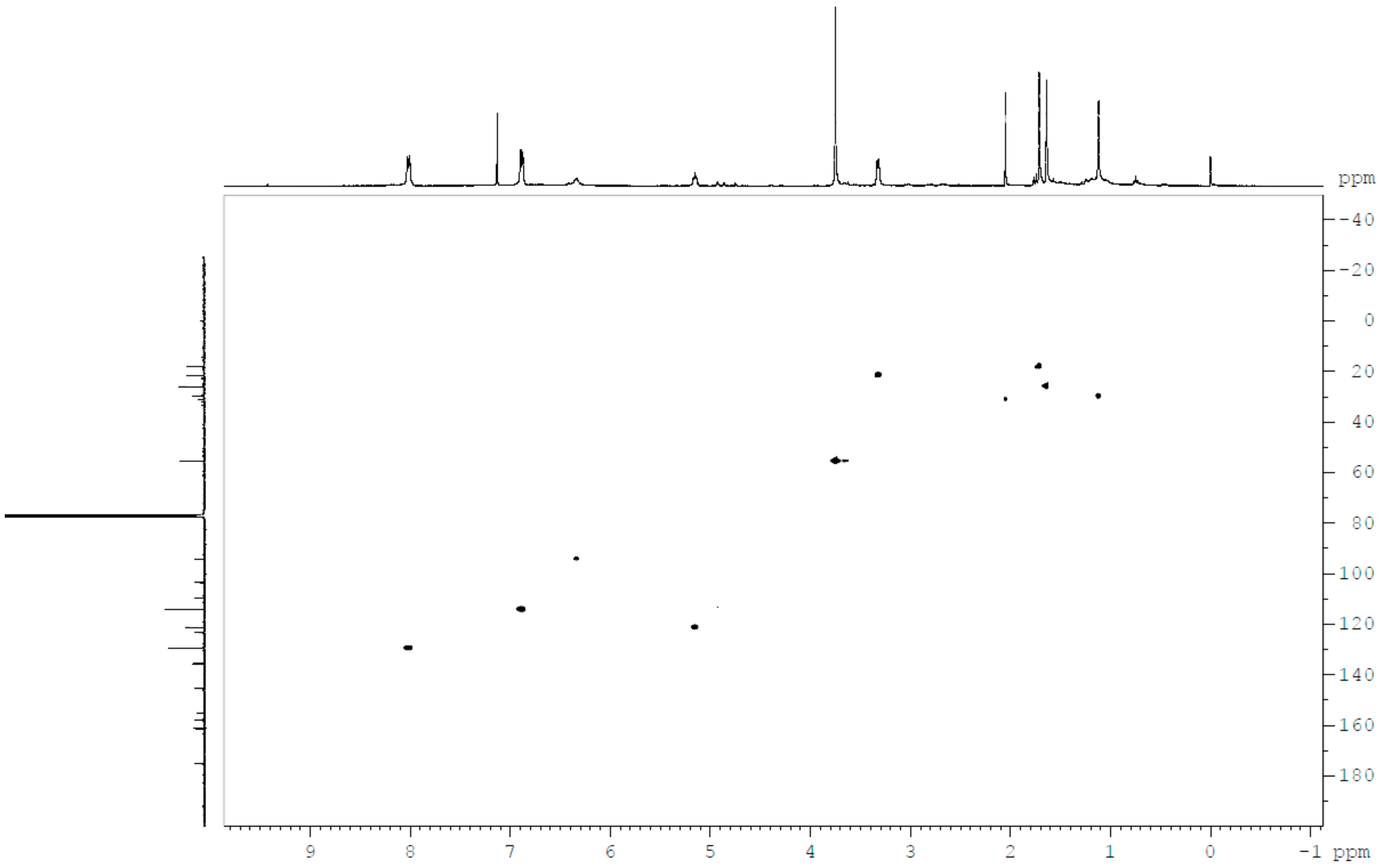

Figure S22. HSQC NMR spectrum of compound **4** ($CDCl_3$, 100 MHz para 13C e 400 MHz para 1H), showing the heteronuclear correlation between H-1'' (δ3,36) e C-1'' (δ161,6)

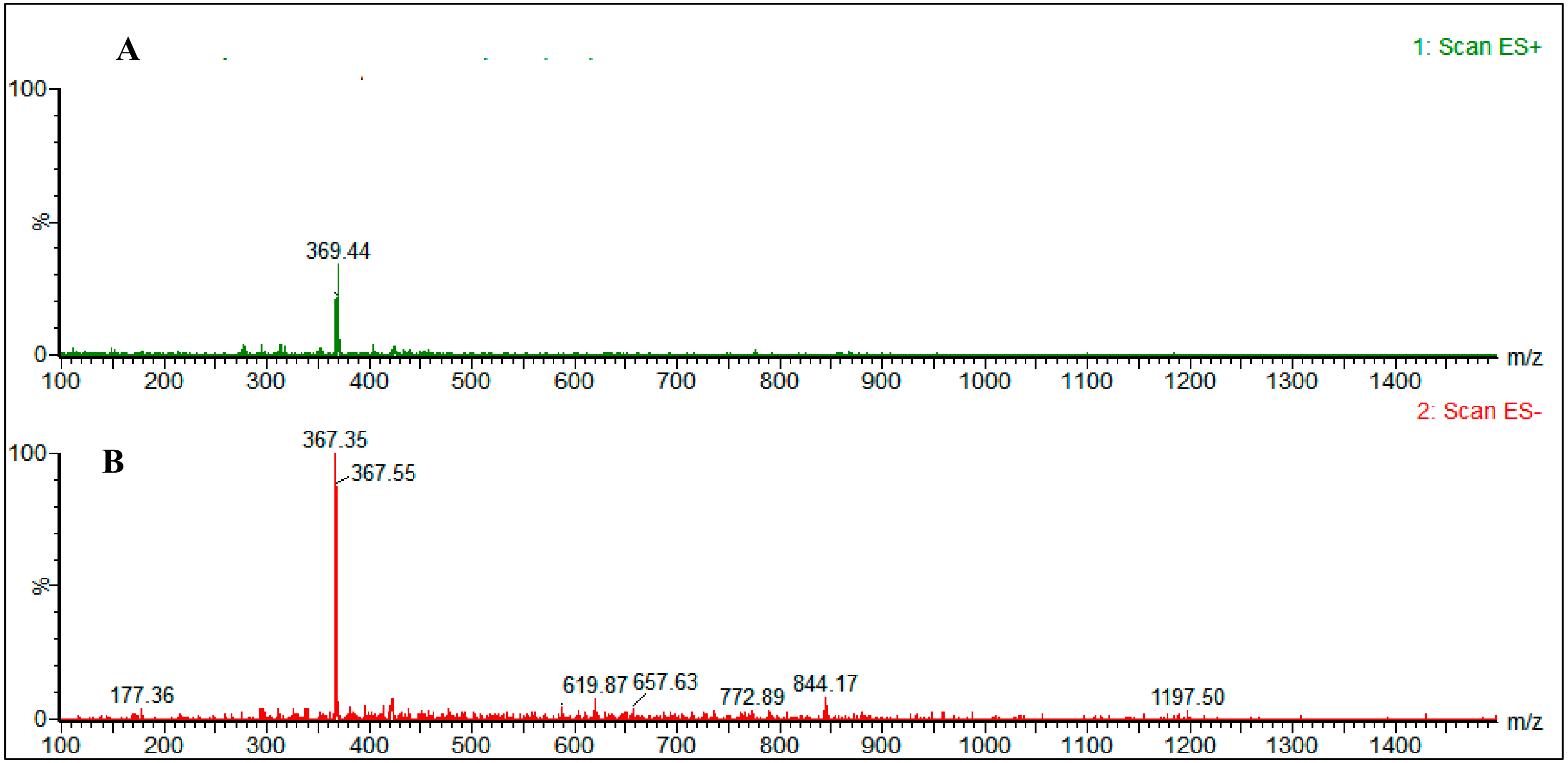

Figure S23. Mass spectra of compound **4** obtained from the ESI-MS experiment in positive mode (A) and negative mode (B)
